# High‐Intensity Interval Training Mitigates Sarcopenia and Suppresses the Myoblast Senescence Regulator EEF1E1

**DOI:** 10.1002/jcsm.13600

**Published:** 2024-09-14

**Authors:** Yaoshan Dun, Wenliang Zhang, Yang Du, Kangling Xie, Yuan Liu, Cui Li, Ling Qiu, Siqian Fu, Thomas P. Olson, Yuqiong Long, Baiyang You, Suixin Liu

**Affiliations:** ^1^ Division of Cardiac Rehabilitation, Department of Physical Medicine & Rehabilitation Xiangya Hospital, Central South University Changsha Hunan China; ^2^ National Clinical Research Centre for Geriatric Disorders, Xiangya Hospital Central South University Changsha Hunan China; ^3^ Division of Preventive Cardiology, Department of Cardiovascular Medicine Mayo Clinic Rochester Minnesota USA; ^4^ Department of Neurology Xiangya Hospital, Central South University Changsha Hunan China

**Keywords:** autophagy, EEF1E1, high‐intensity interval training, sarcopenia, senescence

## Abstract

**Background:**

The optimal exercise regimen for alleviating sarcopenia remains uncertain. This study aimed to investigate the efficacy of high‐intensity interval training (HIIT) over moderate‐intensity continuous training (MICT) in ameliorating sarcopenia.

**Methods:**

We conducted a randomized crossover trial to evaluate plasma proteomic reactions to acute HIIT (four 4‐min high‐intensity intervals at 70% maximal capacity alternating with 4 min at 30%) versus MICT (constant 50% maximal capacity) in inactive adults. We explored the relationship between a HIIT‐specific protein relative to MICT, identified via comparative proteomic analysis, eukaryotic translation elongation factor 1 epsilon 1 (EEF1E1) and sarcopenia in a paired case–control study of elderly individuals (aged over 65). Young (3 months old) and aged (20 months old) mice were randomized to sedentary, HIIT and MICT groups (five sessions/week for 4 weeks; *n* = 8 for each group). Measurements included skeletal muscle index, hand grip strength, expression of atrophic markers Atrogin1 and MuRF1 and differentiation markers MyoD, myogenin and MyHC‐II via western blotting. We examined the impact of EEF1E1 siRNA and recombinant protein on D‐galactose‐induced myoblast senescence, measuring senescence‐associated β‐galactosidase and markers like p21 and p53.

**Results:**

The crossover trial, including 10 sedentary adults (32 years old, IQR 31–32) demonstrated significant alterations in the abundance of 21 plasma proteins after HIIT compared with MICT. In the paired case–control study of 84 older adults (84 years old, IQR 69–81; 52% female), EEF1E1 was significantly increased in those with sarcopenia compared to those without (14.68 [95%CI, 2.02–27.34] pg/mL, *p* = 0.03) and was associated with skeletal muscle index (*R* ^2^ = 0.51, *p* < 0.001) and hand grip strength (*R* ^2^ = 0.54, *p* < 0.001). In the preclinical study, aged mice exhibited higher EEF1E1 mRNA and protein levels in skeletal muscle compared to young mice, accompanied by a lower muscle mass and strength, increased cellular senescence and protein degradation markers and reduced muscle differentiation efficiency (all *p* < 0.05). HIIT reduced EEF1E1 expression and mitigated age‐related muscle decline and atrophy in aged mice more effectively than MICT. Notably, EEF1E1 downregulation via siRNA significantly counteracted D‐galactose‐induced myoblast senescence as evidenced by reduced markers of muscle protein degradation and improved muscle differentiation efficiency (all *p* < 0.05). Conversely, treatments that increased EEF1E1 levels accelerated the senescence process (*p* < 0.05). Further exploration indicated that the decrease in EEF1E1 was associated with increased SIRT1 level and enhanced autophagy.

**Conclusions:**

This study highlights the potential of HIIT as a promising approach to prevent and treat sarcopenia while also highlighting EEF1E1 as a potential intervention target.

## 1 Introduction

The ageing population presents a significant global societal challenge. According to data from the World Health Organization from 2022, the number of individuals aged 60 and older globally will reach 1.4 billion by 2030, with a projected surge to 2.1 billion by 2050. Sarcopenia, a condition among older adults, is characterized by a reduction in skeletal muscle mass and function [1], and is accompanied by issues such as heightened anabolic resistance [2, 3] and protein degradation [4]. This results in functional limitations and a significant decrease in quality of life. Petermann‐Rocha et al. [5] summarized 151 studies and found that the prevalence of sarcopenia in adults over 60 years of age ranges from 10% to 27% and that of severe sarcopenia ranges from 2% to 9%. Sarcopenia significantly increases short‐ and long‐term all‐cause mortality risks [6]. Moreover, it severely impairs the well‐being of older adults, placing a heavy burden on global healthcare systems and society. The pathogenesis of sarcopenia remains unclear, and effective pharmacological interventions are lacking. Currently, the recommended approach involves exercise interventions along with nutritional supplementation [7]. However, the optimal exercise regimen for alleviating sarcopenia has yet to be determined. Thus, it is imperative to develop tailored exercise programmes that are safe and efficacious while elucidating the underlying mechanisms that underpin the benefits of exercise in sarcopenia. This endeavour is of paramount significance in clinical settings.

Recently, attention has been directed towards high‐intensity interval training (HIIT) in clinical exercise therapy. HIIT involves repeated cycles of high‐intensity exercises interspersed with lower intensity recovery periods within a single training session. This approach allows participants to tolerate heightened levels of exercise‐induced physiological stress [8], which is vital for promoting exercise‐related health benefits. A recent prospective observational study suggests that HIIT stimulates skeletal muscles more effectively, as evidenced by greater workload and energy expenditure, while maintaining a steady cardiovascular response, compared with traditional moderate‐intensity continuous training (MICT) [9]. Furthermore, two retrospective observational studies revealed that older patients with myocardial infarction who underwent percutaneous coronary intervention exhibited more enhanced muscle mass and exercise capacity after 12 weeks of HIIT compared to MICT exercise [10, 11]. Corroborative studies, such as those by Bell et al., have evaluated three exercise modalities in older adults and revealed that a bout of acute HIIT significantly increased the sarcoplasmic protein fractional synthetic rate in sedentary older adults [12]. However, despite these advancements, investigations on the comparative effects of HIIT and MICT on sarcopenia are lacking. Furthermore, the mechanisms underlying the augmentation of skeletal muscle content following HIIT remain unclear.

The objective of this study was to employ a methodological blend of a randomized crossover trial (RCT), a sarcopenia‐focused case–control study, experiments on aged mice and myoblast assays to assess the relative effectiveness of HIIT versus MICT in reducing sarcopenia. This study also aimed to identify the key mediators and underlying mechanisms involved.

## 2 Methods

### 2.1 RCT

To profile the plasma proteomic response to HIIT relative to MICT, we used a RCT design to consecutively recruit sedentary adults between 9 May and 31 August 2023. Ten enrolled participants were randomly assigned to the HIIT‐first or MICT‐first groups and subsequently crossed over to the MICT and HIIT groups. The heart rate, blood pressure, rating of perceived exertion and degree of dyspnoea during both MICT and HIIT were continuously monitored. Plasma was collected 5 min before and 15 min after each MICT and HIIT session. Plasma proteomics were profiled using liquid chromatography with tandem mass spectrometry.

The HIIT and MICT protocols were individually prescribed based on the maximum exercise capacity (MEC) of the participants, as indicated by the maximal workload achieved during cardiopulmonary exercise testing. The MICT protocol consisted of a 1‐min warm‐up, followed by training at 50% MEC for 36 min and concluding with a 4‐min cool‐down, resulting in a total exercise duration of 41 min. In contrast, HIIT involved a 5‐min warm‐up, followed by four repetitions of high‐intensity training and a low‐intensity recovery cycle and finished with a 4‐min cool‐down, totalling 41 min. Each HIIT cycle included 4 min of high‐intensity training at 70% MEC, followed by 4 min of low‐intensity training at 30% MEC.

Further details are reported in line with the CONSORT statement specific to RCTs [13] and are provided in the RCT protocol (Data S1). The trial was reviewed and approved by the Ethics Committee and has been registered. All the participants provided written, informed consent.

### 2.2 Sarcopenia Case–Control Study

To scrutinize HIIT‐specific plasma proteins and their relationship with sarcopenia in older adults, we consecutively screened sedentary older adults aged ≥ 60 who underwent annual health examinations between 1 September and 31 October 2023, in Qing‐Yuan community, Changsha, China. Sarcopenia was evaluated according to the 2019 criteria established by the Asian Working Group for Sarcopenia [7]. Fasting blood was collected to perform an enzyme‐linked immunosorbent assay (ELISA) of HIIT‐specific plasma proteins. The concentrations of HIIT‐specific plasma proteins in older adults with and without sarcopenia were compared. Correlations between the concentration of the protein of interest in plasma and skeletal muscle mass and strength were also analysed. Further details are reported in line with the STROBE statement [14] and are provided in Data S2. This study was reviewed and approved by the Ethics Committee.

### 2.3 Animals and Treatment

All procedures involving mice followed the Guidelines for the Use of Live Animals and were approved by the Medicine Animal Welfare Committee. Thirty‐two male C57BL/6J mice were purchased from a certified Laboratory Animal Center. Eight 3‐month‐old mice were assigned to the young control group and subjected to sedentary conditions (young). The 24 20‐month‐old aged mice were randomly divided into the following groups (*n* = 8 per group): sedentary (A‐Sed), MICT (A‐MICT) and HIIT (A‐HIIT).

The HIIT and MICT protocols for mice were modified based on research by Rolim et al. [15]. Mice in the youth and A‐Sed groups were not subjected to any exercise training for the entire duration of the experiment. After 1 week of adaptive feeding, the mice in the A‐MICT and A‐HIIT groups were acclimatized to the treadmill for 3 consecutive days. The mice then underwent an exhaustive running test to obtain MEC, as indicated by the maximal running speed. The training plan was developed according to the MEC of the mice. Briefly, MICT consisted of 40 min of treadmill running at an intensity of 65%–70% MEC. Conversely, the HIIT consisted of high‐low cycles for 40 min, with each cycle comprising 2 min of high‐speed running at an intensity of 85%–90% MEC, followed by 2 min of low‐speed running at an intensity of 30% MEC. The exercise intervention lasted for 4 weeks, with daily sessions performed 5 days per week. An experimental diagram is provided in Data S3. Differences in forelimb grip strength, hanging grid test performance and maximal running speed among the groups were assessed after the intervention. Gastrocnemius weight and body weight; gastrocnemius cross‐sectional area (CSA), measured through wheat germ agglutinin staining; slow muscle and fast muscle proportion, determined by immunofluorescence staining; and skeletal muscle ultrastructure, measured by transmission electron microscopy, were compared between groups. The protein expression of atrophic factors Atrogin1 and MuRF1 and myotube differentiation markers MyoD, myogenin and MyHC‐II were determined by western blotting. *Eef1e1* mRNA expression was assessed by RT‐PCR in several organs identified by The Human Protein Atlas as the main sources of eukaryotic translation elongation factor 1 epsilon‐1 (EEF1E1). Subsequently, skeletal muscle *Eef1e1* mRNA and EEF1E1 protein levels in each group were determined. Additionally, the expression of SIRT and autophagy‐related proteins, including p‐AMPK, AMPK, LC3, p62, p‐ULK and ULK, was determined by western blotting. The reporting of animal studies was in accordance with the ARRIVE guidelines [16]. Further details are provided in Data S3.

### 2.4 In Vitro Experiments

C2C12 mouse myoblasts were incubated with various concentrations of D‐galactose (D‐gal) to induce ageing. The senescence process in myoblasts was evaluated by senescence‐associated β‐galactosidase staining and measuring the levels of senescence markers, such as p21 and p53, and atrophic factors, such as Atrogin1 and MuRF1. To assess differentiation status, the levels of the differentiation marker MyHCII were determined by western blotting and cellular immunofluorescent staining. To investigate the effect of EEF1E1 on autophagy flux, we employed bafilomycin A1 (BafA1), a well‐known inhibitor of autophagic flux.

Senescence myoblasts were incubated with phosphate‐buffered saline (PBS) or recombinant EEF1E1 protein or transfected with the *Eef1e1* plasmid. Following this, EEF1E1 transcription levels and expression and the senescence process of each group were assessed. In addition, SIRT1 expression and autophagic activity in cells were evaluated. The reporting of in vitro experiments was in line with the Checklist for Reporting In Vitro Studies (CRIS) guidelines [17]. Further details are provided in Data S3.

### 2.5 Statistical Analysis

To assess the distribution of continuous variables, we conducted the Shapiro–Wilk test. For continuous variables that followed a normal distribution, we have reported descriptive statistics using mean and standard deviation (mean ± SD). For continuous variables that did not exhibit a normal distribution, we have provided the median and interquartile range (IQR).

In this study, proteomic data were processed using Spectronaut Pulsar, and raw data files were converted to a standard format by local normalization. The data were preprocessed to remove low‐quality spectra and normalize signal intensities across samples. Peptide identification was performed using UniProt with a false discovery rate (FDR) of 0.01 to control for type I errors. Quantitative data analysis was conducted using R software (Version 4.2.0), and peptide intensities were compared across samples using a *t*‐test. Differential expression analysis was performed to identify significantly regulated proteins, with a *p* value < 0.05 and a fold‐change threshold of 1.2 considered statistically significant. Venn analysis was used to visualize shared and unique proteins between the MICT and HIIT groups.

An unpaired two‐tailed *t*‐test was used when comparing the means of two groups. Conversely, an ANOVA with Bonferroni multiple comparison test was used when comparing more than two groups. A Pearson correlation analysis adjusted with age and biological sex was used to assess the association between the two factors. All analyses were performed using the R (Version 4.2.0) software. Statistical significance was set at *p* < 0.05 (two‐sided).

## 3 Results

### 3.1 The HIIT‐Specific Responder EEF1E1 Was Associated With Sarcopenia in Older Adults

**FIGURE 1:**
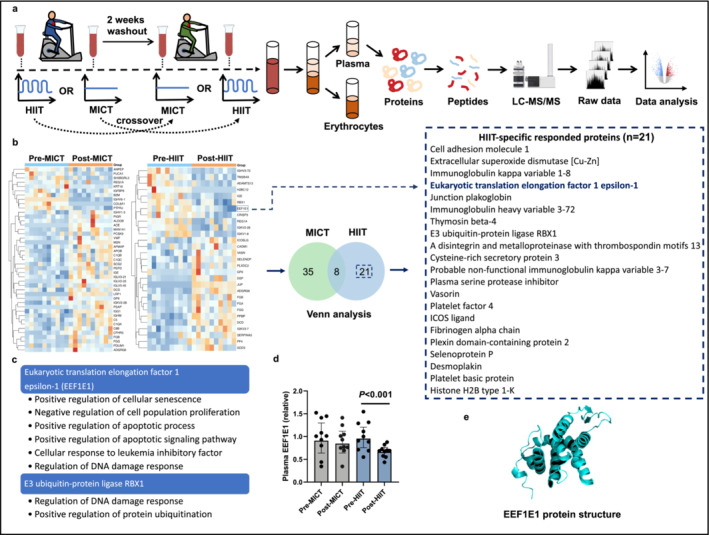
Evaluating plasma protein level changes in sedentary adults following MICT and HIIT. MICT refers to moderate‐intensity continuous training, whereas HIIT denotes high‐intensity interval training. (a) Randomized crossover trial flowchart: Outline of the study design aimed at identifying differentially expressed plasma proteins in sedentary adults. (b) Protein expression heatmaps and Venn diagram: Heatmaps of proteins with differential expression after MICT and HIIT. A Venn diagram is included to show proteins uniquely or commonly affected by MICT and HIIT. (c) EEF1E1‐associated biological processes: Outline of the biological processes related to EEF1E1. (d) EEF1E1 plasma levels: Comparison of the relative plasma concentrations of EEF1E1 before and after MICT and HIIT. (e) EEF1E1 protein structure prediction: Illustration depicting the predicted EEF1E1 protein structure based on UniProt data. Results are shown as the median with the interquartile range (IQR) (*n* = 10). The significance of changes in EEF1E1 levels due to MICT or HIIT was assessed using a paired *t*‐test.

Given that HIIT induces greater skeletal muscle mass in older patients with myocardial infarction than MICT [10, 11], we evaluated specific physiological responses in sedentary adults (Figure S1) and identified distinct circulating blood factors that may influence these responses using a crossover randomized study trial (Figure 1a). A participant flow diagram is shown in Figure S2, and the demographic and clinical information of the participants is presented in Table S1. There were no significant differences in the baseline characteristics between the MICT‐HIIT and HIIT‐MICT groups. Participants underwent cardiopulmonary exercise testing to assess their maximal exercise capacity, which was used to prescribe the individualized MICT and HIIT regimes. The results of the cardiopulmonary exercise testing are provided in Table S2. The relative quantities of soluble proteins in the plasma of inactive adults were quantified using liquid chromatography coupled with tandem mass spectrometry, both before and after engaging in HIIT and MICT.

In sedentary adults, the abundance of 29 proteins changed significantly following HIIT, with 21 specific responders compared to MICT (Figure 1b). These factors include EEF1E1, cell adhesion molecule 1 (CADM1), extracellular superoxide dismutase [Cu‐Zn] (SOD3), immunoglobulin kappa variable 1‐8 (IGKV1‐8), junction plakoglobin (JUP), immunoglobulin heavy variable 3‐72 (IGHV3‐72), thymosin beta‐4 (TMSB4X), E3 ubiquitin‐protein ligase RBX1 (RBX1), a disintegrin and metalloproteinase with thrombospondin motifs 13 (ADAMTS13), cysteine‐rich secretory protein 3 (CRISP3), probable non‐functional immunoglobulin kappa variable 3‐7 (IGKV3‐7), plasma serine protease inhibitor (SERPINA5), vasorin (VASN), platelet factor 4 (PF4), ICOS ligand (ICOSLG), fibrinogen alpha chain (FGA), plexin domain‐containing protein 2 (PLXDC2), selenoprotein P (SELENOP), desmoplakin (DSP), platelet basic protein (PPBP) and histone H2B type 1‐K (H2BC12). Table S3 provides information on the proteins that were expressed differently. According to information obtained from the Human Protein Atlas [18], 57% of HIIT‐specific responders are predominantly localized to or expressed in the skeletal muscle. Functional analysis of these factors using UniProt [19], a search tool for protein sequences and functional information, revealed that EEF1E1 and RBX1 play significant roles in the DNA damage response and cellular senescence (Figure 1c). We subsequently investigated EEF1E1, a modulator of the ATM response to DNA damage that is involved in the regulation of cellular senescence [20] and has not been previously linked to sarcopenia. After engaging in HIIT, sedentary young adults exhibited a significant reduction in plasma EEF1E1 levels; however, these levels remained unchanged following MICT (Figure 1d). The structure of EEF1E1 predicted using the AlphaFold 3 is shown in Figure 1e.

**FIGURE 2:**
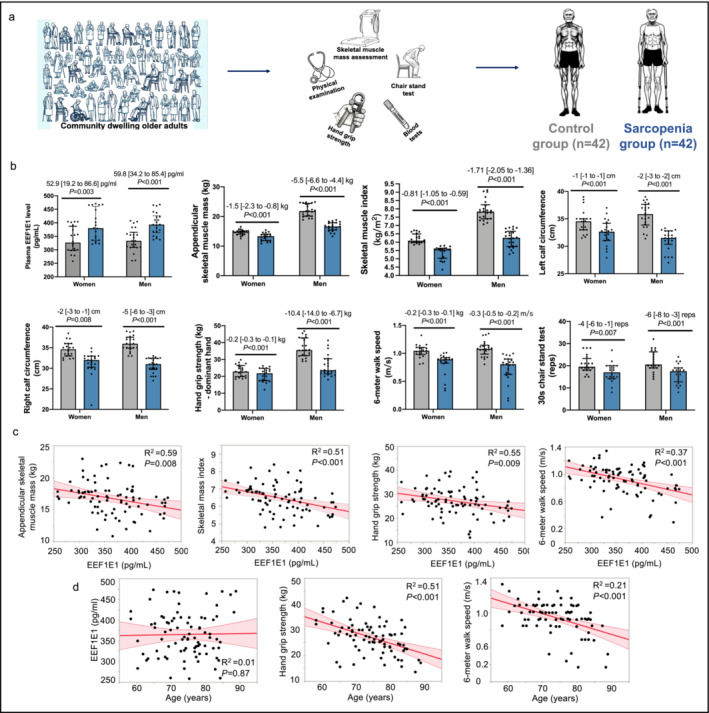
Linking EEF1E1 with sarcopenia in older adults. EEF1E1 represents the eukaryotic translation elongation factor 1 epsilon 1. This figure summarizes results from a sarcopenia case–control study in a population of community‐dwelling older adults (*n* = 84). (a) Study flowchart: Illustration of the design and progression of the sarcopenia case–control study. (b) Comparisons of EEF1E1 plasma levels, appendicular skeletal muscle mass, SMMI values, left calf circumference measurements, right calf circumference measurements, handgrip strength assessment, 6‐m walk test speeds and 30‐s chair stand test performance between the control and sarcopenia groups. Comparative analysis: An independent *t*‐test was used for comparing the difference in outcomes between the control and sarcopenia groups. (c) Correlation analysis: A Pearson correlation, adjusted for biological sex and age, was used to assess the relationship between EEF1E1 levels and appendicular skeletal muscle mass, skeletal muscle mass index, handgrip strength and 6‐m walk test speed. (d) Correlation analysis: A Pearson correlation, adjusted for biological sex, was used to assess the relationship between age and EEF1E1 levels, hand grip strength and 6‐m walk speed.

In a separate study analysing sarcopenia cases and controls (Figure 2a), it was observed that older adults suffering from sarcopenia exhibited higher plasma levels of EEF1E1 compared to their counterparts without sarcopenia (Figure 2b). Furthermore, in the older adults, irrespective of sarcopenia status, a notable association was found between heightened levels of EEF1E1 in the plasma and a decrease in skeletal muscle mass, muscular strength and physical performance (Figure 2c). Additionally, although no significant correlation was found between age and EEF1E1 levels, a significant association was observed between increased age and reductions in hand grip strength and 6‐m walk speed (Figure 2d). These data implicated EEF1E1 as a HIIT‐specific circulating blood target in older adults with potential relevance to sarcopenia. The characteristics of the participants in the sarcopenia case–control study are presented in Table S4.

### 3.2 HIIT Mitigates EEF1E1 Expression and Counteracts the Decline in Muscle Mass and Strength in Aged Mice

**FIGURE 3:**
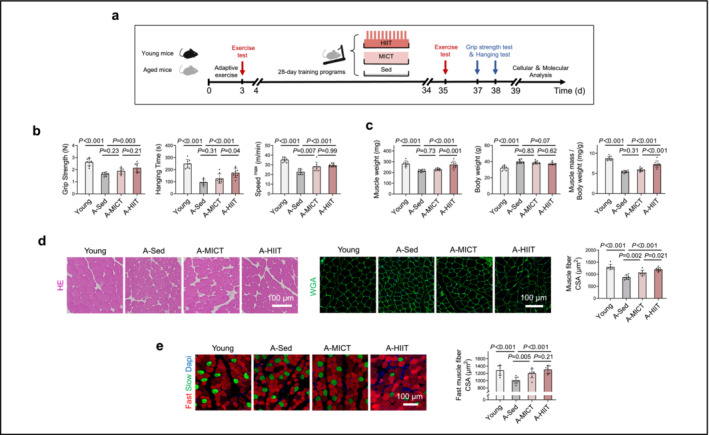
HIIT counteracts muscle mass and strength loss in aged mice. (a) Animal protocol: The diagram illustrates the protocol used. Aged mice (20 months old) were divided into three groups: sedentary (A‐Sed), moderate‐intensity continuous training (A‐MICT) and high‐intensity interval training (A‐HIIT). These groups underwent respective 4‐week training programs. Young mice (3 months old) served as the normal control group (Young). (b) Physical performance tests: The grip strength, hanging time and maximum running speed of the mice were evaluated (*n* = 8). (c) Body and muscle weight assessment: body weight in grams, muscle complex mass (gastrocnemius, plantaris and soleus) of a single leg in milligrams and the ratio of muscle complex mass to body weight were assessed (*n* = 8). (d) myofibre analysis: The cross‐sectional area (CSA) of gastrocnemius myofibres was measured using haematoxylin–Eosin (HE) and wheat germ agglutinin (WGA) staining (scale bar = 100 μm, *n* = 8). (e) Fibre type identification: Fast (red) and slow (green) muscle fibres were identified using stained with immunofluorescence staining (scale bar = 100 μm). The CSA of fast muscle fibres was analysed (*n* = 8). Data are presented as the mean ± SD. An independent two‐tailed *t*‐test was used for comparisons between the Young and A‐Sed groups. An ANOVA with Bonferroni multiple comparisons test was applied for the A‐Sed, A‐MICT and A‐HIIT groups.

We assessed the structural and functional changes in skeletal muscles due to senescence by comparing aged and young mice (Figure 3a). Aged mice exhibited reduced grip strength, hanging time and running speed (Figure 3b). Additionally, there were reductions in gastrocnemius muscle weight and muscle mass in proportion to body weight (Figure 3c), a decrease in muscle fibre cross‐sectional area (Figure 3d) and a lower percentage of fast‐twitch muscle fibres (Figure 3e), despite the aged mice having a higher total body weight (Figure 3c).

**FIGURE 4:**
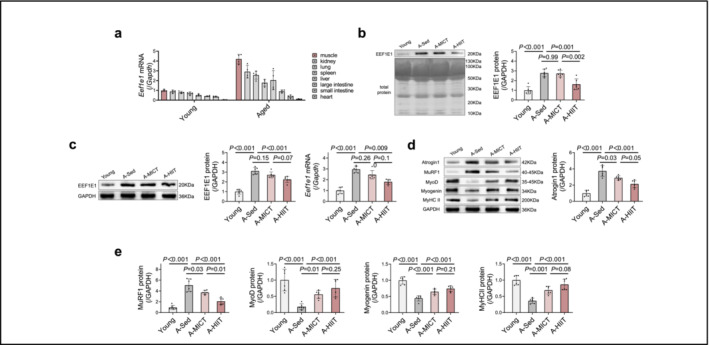
HIIT mitigates EEF1E1 expression and atrophy in aged mice. (a) *Eef1e1* mRNA expression in young and aged mice: *Eef1e1* mRNA levels in various main organs were assessed (*n* = 4). (b) Plasma EEF1E1 protein levels: Plasma EEF1E1 protein expression was determined (*n* = 6). (c,d) Muscle EEF1E1 protein and *Eef1e1* mRNA expression: The EEF1E1 protein and *Eef1e1* mRNA levels in each group were measured (*n* = 6). (e) Protein expression of atrophy and differentiation markers: The expression levels of the atrophic factors Atrogin1 and MuRF1 and the differentiation markers MyoD, myogenin and MyHC II were determined (*n* = 6). Data are presented as the mean ± SD. An independent two‐tailed *t*‐test was used for comparisons between the Young and A‐Sed groups. An ANOVA with Bonferroni multiple comparison test was applied for the A‐Sed, A‐MICT and A‐HIIT groups.

In order to identify the potential origin of exercise‐induced systemic EEF1E1, we conducted an assessment of *Eef1e1* mRNA expression across various tissues in young mice, including muscles, kidneys, lungs, spleen, liver, large intestine, small intestine and hearts (Figure 4a). Our findings revealed the greatest level of *Eef1e1* mRNA expression in the skeletal muscle.

Notably, aged mice exhibited an increase in EEF1E1 plasma levels (4b) and higher mRNA and protein expression of EEF1E1 in skeletal muscles, as depicted in Figure 4c,d. Additionally, these mice displayed elevated levels of cellular senescence markers, including p16, p21, p53 and beta‐galactosidase, in the skeletal muscle compared to younger mice (Figure S3). We observed increased levels of markers of muscle protein degradation, including Atrogin1 and MuRF1, and decreased muscle differentiation efficiency, as indicated by the downregulation of MyoD, Myogenin and MyHCII in aged mice (Figure 4e).

To examine the effects of HIIT and MICT on aged skeletal muscle, aged mice underwent treadmill training for a duration of 4 weeks, administered by experienced laboratory personnel. During this period, the mice participated in either HIIT or MICT sessions. Meanwhile, a group of sedentary control mice of the same age were provided with nesting material. The findings indicated that HIIT resulted in a significant decrease in plasma EEF1E1 levels (Figure 4b), along with reduced EEF1E1 mRNA and protein expression in the skeletal muscle of aged mice (Figure 4c,d). Conversely, such effects were not observed with MICT, as depicted in Figure 4b–d. Moreover, HIIT improved skeletal muscle structure, as evidenced by increased muscle mass relative to body weight, fast muscle fibres and total cross‐sectional area, whereas MICT only increased fast muscle fibres (Figure 3b–d). Functional enhancements following HIIT were also observed, including increased grip strength, longer hanging times and faster running speeds compared with sedentary controls (Figure 3b–d). In contrast, MICT increased running speed only (Figure 3b). Both HIIT and MICT exhibited protective effects against muscle protein degradation, as demonstrated by the downregulation of Atrogin1 and MuRF1 (Figure 4e). Additionally, both training modalities enhanced muscle differentiation efficiency, which was marked by increased MyoD, myogenin and MyHC‐II expression in aged mice. Notably, HIIT was more effective than MICT in promoting muscle differentiation (Figure 4e).

### 3.3 EEF1E1 Downregulation Slows Skeletal Muscle Cell Senescence, Whereas EEF1E1 Overexpression Accelerates It

**FIGURE 5:**
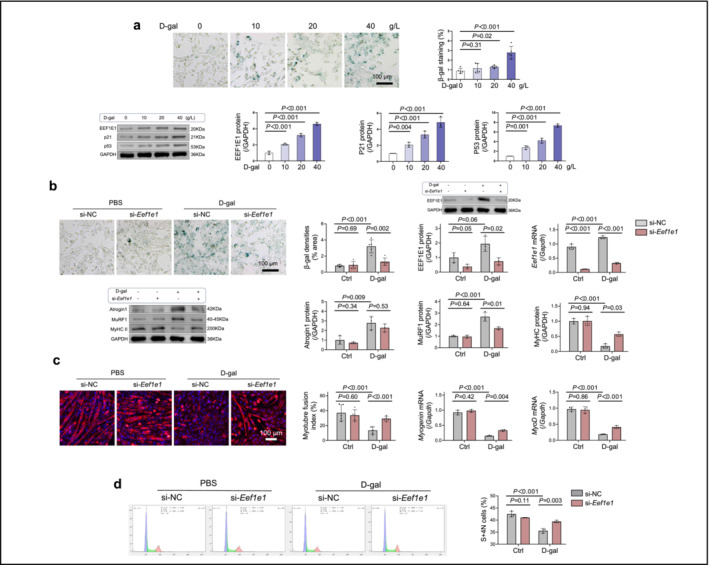
*Eef1e1* suppression ameliorates measures of senescence, reduces protein degradation and accelerates fusion in skeletal muscle cells. (a) SA‐β‐galactosidase staining in myoblasts: Myoblasts incubated with varying concentrations of D‐galactose (D‐gal) underwent SA‐β‐ galactosidase staining. Analysis was performed using a scale bar of 100 μm (*n* = 5). Protein expression analysis: The protein expression levels of EEF1E1, p21 and p53 were determined (*n* = 3). (b) Effects of *Eef1e1*‐siRNA transfection: Cells were transfected with *Eef1e1*‐siRNA (si‐*Eef1e1*) or control‐siRNA (si‐NC) in the presence of D‐gal or phosphate‐buffered saline (PBS). SA‐β‐Galactosidase staining assessment: Staining was performed and analysed (scale bar = 100 μm, *n* = 5). The protein and mRNA expression levels of EEF1E1 were measured (*n* = 3). Atrophy markers: The protein expression of Atrogin1, MuRF1 and MyHC II was determined (*n* = 3). (c) MyHCII immunofluorescence: Cellular immunofluorescent staining of MyHCII was conducted post‐differentiation into myotubes (scale bar = 100 μm, *n* = 3). Myotube index: The ratio of nuclei in myotubes to total nuclei was analysed as an index of myotube formation (*n* = 3). Myogenic factors: mRNA levels of myogenin and MyoD were measured (*n* = 3). (d) Cell cycle analysis: The cell cycle was assessed using flow cytometry (*n* = 3). Data are presented as the mean ± SD. ANOVA with Bonferroni multiple comparison test was applied to (a). For (b,c), ANOVA and independent two‐tailed *t*‐tests were used as deemed appropriate.

In order to explore the effect of EEF1E1 on skeletal muscle cell senescence, we initially employed D‐gal to induce senescence in myoblasts. The D‐gal‐induced treatment led to the appearance of senescent cell traits in myoblasts, as evidenced by increased β‐galactosidase activity revealed through β‐galactosidase staining and elevated levels of the senescence biomarkers p21 and p53 (Figure 5a). Additionally, we observed a dose‐dependent relationship between D‐gal concentration and myoblast senescence (Figure 5a). D‐gal also elevated the expression levels of Atrogin‐1 and MuRF1, markers of cellular protein degradation (Figure 5b). Concurrently, there were a decrease in the myotube fusion index (the ratio of myotube nuclei to total nuclei) and a reduction in mRNA levels of Myogenin and MyoD, suggesting compromised muscle differentiation efficiency in myoblasts (Figure 5c). Additionally, D‐gal reduced the percentage of proliferative S‐4N cells, a key indicator of cell proliferation (Figure 5d).

We administered *Eef1e1* siRNA to myoblasts, both untreated and treated with D‐gal, to downregulate EEF1E1 expression. The results showed that the siRNA treatment significantly reduced both mRNA and protein levels of EEF1E1 (Figure 5b). Furthermore, *Eef1e1* siRNA countered the D‐gal‐induced increase in Atrogin‐1 and MuRF1 expression, the decrease in the myotube fusion index and the reduction in MyHC protein and Myogenin and MyoD mRNA levels (Figure 5b,c). Additionally, *Eef1e1* siRNA treatment led to an increase in the percentage of proliferative S‐4N cells (Figure 5d). These results suggest that *Eef1e1* siRNA mitigated the adverse effects of D‐gal on cellular protein degradation and differentiation efficiency in myoblasts.

**FIGURE 6:**
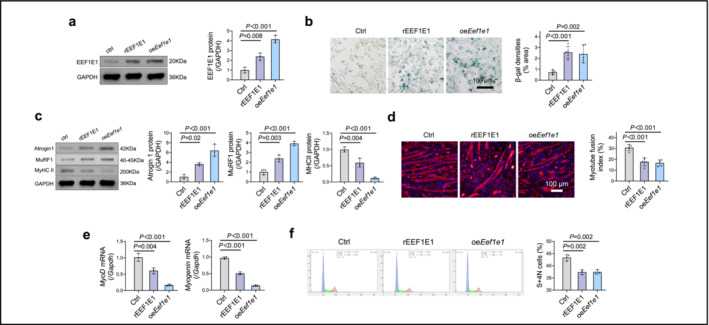
*Eef1e1* overexpression and supplementation exacerbate measures of senescence, promote protein degradation and inhibit fusion in skeletal muscle cells. (a) EEF1E1 protein expression in myoblasts: Myoblasts were treated with phosphate‐buffered saline (PBS), recombinant EEF1E1 protein (rEEF1E1) or transfected with an *Eef1e1* plasmid (*oeEef1e1*) and the EEF1E1 protein expression was determined (*n* = 3). (b) SA‐β‐Galactosidase staining was performed on treated myoblasts; scale bar = 100 μm (*n* = 5). (c) Protein expression: Determination of protein levels of Atrogin1, MuRF1 and MyHCII (*n* = 3). (d) MyHCII immunofluorescence was performed post‐differentiation into myotubes; scale bar = 100 μm. The ratio of nuclei in myotubes to total nuclei was analysed (*n* = 5). (e) mRNA expression: mRNA levels of *MyoD* and *Myogenin* (*n* = 3). (f) Cell cycle analysis was performed using flow cytometry (*n* = 3). Data are expressed as the mean ± SD. Statistical analysis was performed using ANOVA with Bonferroni multiple comparison test.

Conversely, when myoblasts were subjected to treatment with recombinant EEF1E1 or overexpressed EEF1E1 (Figure 6a), they exhibited pronounced senescent characteristics. This was indicated by enhanced β‐galactosidase activity (Figure 6b), increased levels of the cell protein degradation markers Atrogin1 and MuRF1 and a reduced level of the muscle differentiation efficiency marker, MHCII (Figure 6c). Concurrently, there was a decline in muscle differentiation efficiency, evidenced by a lower myotube fusion index (Figure 6d) and decreased mRNA levels of myogenin and MyoD (Figure 6e). Additionally, increased expression of EEF1E1 led to a decreased percentage of proliferative S‐4N cells, which serve as an indicator of cell proliferation, as revealed through flow cytometry–based cell cycle analysis (Figure 6f).

### 3.4 Sarcopenia in Mice and D‐Gal‐Induced Myoblast Senescence Are Associated With Decreased SIRT1 Level and Autophagy Activity

**FIGURE 7:**
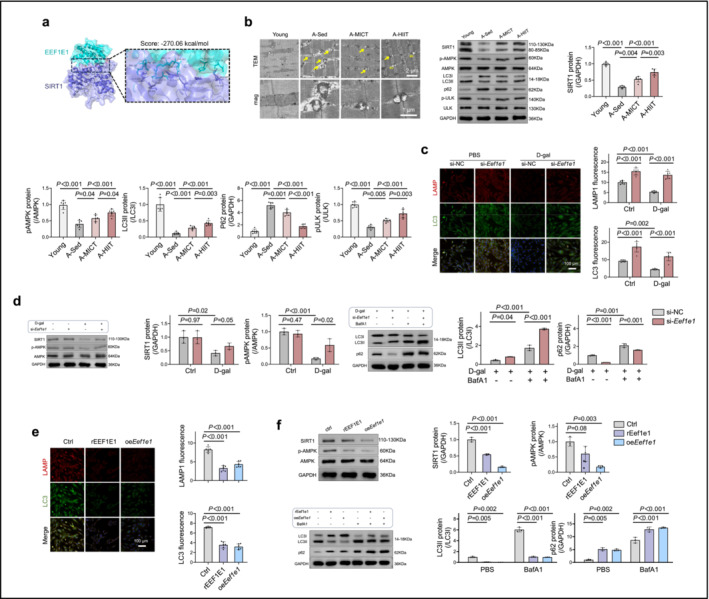
Correlation between sarcopenia in mice and D‐gal‐induced myoblast senescence with decreased SIRT1 levels and autophagy activity. (a) Protein–protein docking: Illustration of the interaction between EEF1E1 and SIRT1. (b) Experimental groups and training protocols: Aged mice were categorized into sedentary (A‐Sed), moderate‐intensity continuous training (A‐MICT) and high‐intensity interval training (A‐HIIT) groups, each undergoing a 4‐week training programme. Young mice served as the normal control group (Young). Electron microscopy of skeletal muscle samples: Transmission electron microscope images highlighting damaged mitochondria (yellow arrows) and autophagy vesicles (av); scale bars = 2 or 1 μm. Protein expressions of SIRT1, pAMPK, AMPK, LC3I, LC3II, p62, pULK and ULK in muscle tissues were analysed (*n* = 6). (c) Cellular immunofluorescence in muscle cells: Following transfection with *Eef1e1*‐siRNA (si‐*Eef1e1*) or control‐siRNA (si‐NC) and treatment with D‐gal or phosphate‐buffered saline (PBS), cells were stained for LAMP and LC3; scale bar = 100 μm (*n* = 6). (d) Protein expression analysis in transfected cells: Levels of SIRT1, pAMPK and AMPK were determined (*n* = 3). Then, following transfection with si‐*Eef1e1* or si‐NC and treatment with phosphate‐buffered saline (PBS) or bafilomycin A1 (BafA1), LC3I, LC3II and p62 protein expression were determined (*n* = 3). (e) Cellular immunofluorescence post‐treatment: Following incubation with PBS, BafA1, rEEF1E1 protein or *Eef1e1* plasmid transfection, LAMP and LC3 staining were conducted; scale bar = 100 μm (*n* = 6). (f) Protein expression post‐treatment: Quantification of SIRT1, pAMPK, AMPK, LC3I, LC3II and p62 levels (*n* = 3). Data are presented as the mean ± SD. For (b), an independent two‐tailed *t*‐test was used for comparisons between the Young and A‐Sed groups. For (c–f), ANOVA with Bonferroni multiple comparison test was used for the comparisons.

Sirtuins, such as SIRT1, are integral to processes that are central to muscle ageing and the pathology of sarcopenia [21]. Subsequently, protein–protein docking was conducted to explore the correlation between SIRT1 and EEF1E1. Based on the −270.06 kcal/mol docking score (Figure 7a), we speculated that SIRT1 serves as a target of EEF1E1. We also assessed SIRT1 expression and its downstream autophagy pathways in the skeletal muscles of aged mice and observed a reduction in SIRT1 levels in aged skeletal muscles. This reduction was paralleled by a decrease in autophagic activity, marked by diminished conversion of LC3I to LC3II, which is crucial for autophagosome formation. Additionally, there were a significant decrease in phosphorylated ULK, a primary initiator of autophagy and the accumulation of p62, a cargo protein typically degraded during autophagy, indicating reduced autophagic flux in aged muscle tissue. Both MICT and HIIT effectively countered age‐related suppression of SIRT1 and autophagy, with HIIT demonstrating superior efficacy (Figure 7b).

Furthermore, silencing *Eef1e1* using siRNA reversed the D‐gal‐induced inhibition of SIRT1 expression and autophagy in myoblasts, as demonstrated by increased AMPK phosphorylation, enhanced conversion of LC3I to LC3II, lowered p62 levels and increased autophagosome formation, as revealed by cellular immunofluorescence staining of autophagy markers (Figure 7c,d). In contrast, recombinant EEF1E1 treatment and *Eef1e1* overexpression suppressed SIRT1 levels and autophagy in these cells (Figure 7e,f).

Additionally, we examined the impact of si*Eef1e1*, rEef1e1 and oe*Eef1e1* on autophagy markers in both the presence and absence of BafA1. In both the presence and absence of BafA1, si*Eef1e1* consistently increased LC3II expression, indicating that si*Eef1e1* activates autophagy flux (Figure 7d). Conversely, rEef1e1 and oe*Eef1e1* inhibited autophagy flux under both conditions (Figure 7f).

## 4 Discussion

This study demonstrated an association between the HIIT‐specific responder EEF1E1 to sarcopenia through a RCT involving sedentary adults and a case–control study involving older adults living in the community. Further, it was demonstrated that HIIT effectively reduced EEF1E1 levels, leading to improvements in age‐related structural and functional impairments in the skeletal muscles of aged mice. Additionally, *Eef1e1* suppression using siRNA effectively ameliorated the senescence process and dysfunction in skeletal muscle cells, whereas recombinant EEF1E1 treatment or *Eef1e1* overexpression accelerated the senescence. These results highlight the potential of HIIT as a promising strategy for preventing and treating sarcopenia and underscore EEF1E1 as a promising target for intervention.

HIIT has emerged as a prominent topic in clinical exercise treatment strategies. Robinson et al. [22] demonstrated that HIIT increased skeletal muscle mitochondrial function and muscle strength. Additionally, in a study conducted by Blue et al. [23], the impact of a 3‐week HIIT intervention on muscle size and mass was examined in overweight and obese individuals. Their findings demonstrated substantial enhancements in cross‐sectional muscle area and noteworthy changes in muscle characteristics and remodelling. Furthermore, Bell et al. reported that HIIT significantly elevated the sarcoplasmic protein fractional synthesis rate in sedentary men compared to resistance exercise and MICT [12]. Our findings indicate that, compared to MICT, HIIT effectively improves age‐related structural and functional impairments in the skeletal muscles of aged mice. These results suggest that HIIT may serve as a supplementary exercise regimen when combined with other evidence‐based exercise approaches, such as resistance exercise [7] and the circuit training [24], as well as appropriate nutritional support, to prevent and treat sarcopenia.

The mechanisms by which HIIT promotes increased skeletal muscle content remain elusive. A recent single‐arm trial involving eight male adults examined the effects of HIIT on the proteome of human skeletal muscles and revealed the responses of 3168 proteins [25]. However, this study lacked comparative information with MICT and association analyses between key responders and sarcopenia. In our study, we discovered 21 plasma responders specific to HIIT compared with MICT using comparative proteomics in a RCT. Furthermore, our research confirmed a negative association between EEF1E1 levels in the skeletal muscle and sarcopenia through animal and in vitro experiments, consistent with previous reports that identified skeletal muscle as the primary source of circulating EEF1E1 [18]. Further studies are required to identify other HIIT‐specific responders in various tissues.

EEF1E1 plays a pivotal role within the eukaryotic translation elongation factor 1 complex, which aids in the transport of aminoacyl tRNAs to ribosomes, facilitating the process of protein synthesis. Previous experiments in mice have shown that EEF1E1 plays a role in inhibiting cell population proliferation, promoting the apoptotic signalling pathway and related processes, as well as enhancing the DNA damage response and signal transduction through p53 class mediators [26]. Additionally, EEF1E1 may be associated with the positive regulation of cellular senescence, as suggested by the sequence orthology. In the present study, case–control research has demonstrated that plasma levels of EEF1E1 are correlated with sarcopenia, skeletal muscle index and muscle strength. Observations from a RCT involving sedentary adults, as well as experimental interventions in 20‐month‐old aged mice, suggest that HIIT may be a potential intervention for regulating EEF1E1 levels. In vitro experiments demonstrated that downregulation of EEF1E1 via siRNA significantly counteracted the senescence induced in myoblasts by D‐galactose, whereas treatments that increased EEF1E1 levels accelerated the senescence process. Further exploration of the regulatory pathways using cellular model suggested that the decrease in EEF1E1 was associated with an increase in SIRT1 levels and enhanced autophagy.

SIRT1 functions as a deacetylase. SIRT1 promotes autophagy by deacetylating key autophagy‐related proteins and enhancing their activity, which is essential for maintaining cellular homeostasis [27]. A recent study reported that autophagy plays a role in various exercise interventions designed to mitigate skeletal muscle atrophy in aged rats [28]. Thus, SIRT1 and autophagy are believed to play a protective role by promoting the clearance of damaged cellular components, potentially delaying or resisting the onset of sarcopenia. Our findings only suggest increased SIRT1 levels and enhanced autophagy are associated with alleviation of myoblast senescence induced by D‐gal. Further studies are warranted to demonstrate the direct causal relationships between SIRT1 and autophagy, as well as SIRT1 and EEF1E1, and their roles in sarcopenia and cellular senescence. Moreover, investigating their interaction with other established muscle degradation regulators such as FoxOs [29] represents a promising avenue for study.

## 5 Limitations

Despite the notable results of this study, it comes with certain limitations. Primarily, the effects of MICT and HIIT on plasma proteomic responses were assessed in sedentary young adults and sarcopenia in aged mice, but not directly in older adults with sarcopenia, potentially limiting the applicability of our findings. This underlines the need for additional independent clinical trials in older adult populations with sarcopenia, and future investigations should consider the effect of biological sex on the results. Furthermore, although siRNA, overexpression and recombinant EEF1E1 interventions aided in validating the role of EEF1E1 in sarcopenia, further research, possibly using EEF1E1 knockout mice, would be advantageous. Additionally, although we identified plasma EEF1E1 as a potential marker of sarcopenia and observed its ability to influence myoblast senescence, SIRT1 and autophagy when introduced into cell media, the present study did not elucidate how EEF1E1 interacts with SIRT1 and autophagy, nor did it clarify the regulatory relationship between SIRT1 and autophagy. Further research is required to investigate the underlying mechanisms. Moreover, instead of suggesting direct applicability to elderly human populations, the findings from these experiments offer potential insights for elderly humans. These results will require validation through clinical trials before any firm conclusions can be drawn.

## 6 Conclusion

This study revealed the viability of HIIT as an effective strategy for the prevention and management of sarcopenia and underscored the potential of EEF1E1 as a candidate for therapeutic targeting.

## Ethics Statement

This manuscript contains studies that have been rigorously reviewed and approved by the Ethics Committee of Xiangya Hospital, Central South University, ensuring adherence to the ethical standards of the 1964 Declaration of Helsinki and its later amendments. Specifically, the RCT (approval number 2023030379, registered at Chictr.org.cn under ChiCTR2300071797) and the case–control study (approval number 202305365) involved human participants who provided informed consent, with reports aligning with the CONSORT and STROBE statements respectively. Additionally, our animal study involving mice adhered to live animal use guidelines and was approved by the Medicine Animal Welfare Committee of Xiangya Medical School (approval ID: CSU‐2023‐0155), with reporting in accordance with the ARRIVE guidelines.

## Conflicts of Interest

The authors declare no conflicts of interest.

## Supporting information


**Data S1** Supporting Information

**Data S2** Supporting Information

**Data S3** Supporting Information

**Table S1** Characteristics of Participants from the Randomized Crossover Trial (*N* = 10).
**Table S2.** Characteristics of Cardiopulmonary Exercise Testing (N = 10).
**Table S3.** Details of Differentially Expressed Plasma Proteins after MICT and HIIT.
**Table S4.** Characteristics of Participants in the Sarcopenia Case–Control Study.
**Figure S1.** Comparative Physiological Responses to High‐Intensity Interval Training (HIIT) and Moderate‐Intensity Continuous Training (MICT) in Sedentary Adults. MEC indicates maximal exercise capacity. Panels (A‐C) illustrate the responses in exercise intensity, heart rate, systolic blood pressure, and perceived exertion and dyspnea during HIIT; while Panels (D‐F) depict these responses during MICT.
**Figure S2.** Participant Flow Diagram for the Randomized Crossover Trial. MICT refers to moderate‐intensity continuous training, while HIIT denotes high‐intensity interval training.
**Figure 3.** Expression of Senescence Markers of Muscle in Aged Mice. Protein expressions of P16, P21, P53, and beta‐galactosidase (GLB1) in muscle tissues were analyzed (*n* = 3). Data are presented as the mean ± SD. An independent two‐tailed t‐test was used for comparisons between the young and aged groups.

## Data Availability

The proteomic data from this study are available in the Integrated Proteome Resources (iProX) database. These can be accessed via the following link: https://www.iprox.cn/page/PSV023.html;?url=1706544900250wn9d using the password: JmD3. All other datasets generated and/or analysed during this study are accessible in the Figshare repository through this link: https://figshare.com/s/53a20dc99d62fcd5a1a2.
