# Supplementary material for: High‐Intensity Interval Training Mitigates Sarcopenia and Suppresses the Myoblast Senescence Regulator EEF1E1
Source: J Cachexia Sarcopenia Muscle. 2024 Sep 14;15(6):2574–85. doi: 10.1002/jcsm.13600 (PMC11634493; doi:10.1002/jcsm.13600)
Supplement: Supplementary file 1 — Data S1 Supporting Information [file JCSM-15-2574-s002.docx]

**Supplementary Document 1: Randomized crossover trial protocol**

**CONTENTS**

[1. GENERAL INFORMATION 3](#_Toc155594492)

[1.1 Trial registration 3](#_Toc155594493)

[2. SYNOPSIS 3](#_Toc155594494)

[3. INTRODUCTION 8](#_Toc155594495)

[3.1 Rationale 8](#_Toc155594496)

[3.2 Objectives 9](#_Toc155594497)

[4. METHODS 11](#_Toc155594498)

[4.1 Trial design 11](#_Toc155594499)

[4.2 Setting 11](#_Toc155594500)

[4.3 Selection of trial participants 11](#_Toc155594501)

[4.4 Sample size 13](#_Toc155594502)

[4.5 Interventions 14](#_Toc155594503)

[4.6 Outcomes 14](#_Toc155594504)

[4.7 Statistical methods 15](#_Toc155594505)

[4.8 Participant timeline 15](#_Toc155594506)

[4.9 Recruitment 16](#_Toc155594507)

[4.10 Assignment and allocation of intervention 16](#_Toc155594508)

[4.11 Blinding 16](#_Toc155594509)

[4.12 Data management 17](#_Toc155594510)

[5. INDIVIDUAL TRIAL PROCEDURE AND OUTCOMES ASSESSMENT 17](#_Toc155594511)

[6. ETHICAL AND LEGAL ASPECTS 28](#_Toc155594512)

[6.1 Ethical approval 28](#_Toc155594513)

[6.2 Confidentiality 28](#_Toc155594514)

[6.3 Others 28](#_Toc155594515)

[7. Informed Consent Form 32](#_Toc155594516)

[8. CONSORT Checklist 34](#_Toc155594517)

# GENERAL INFORMATION

## Trial registration

Chinese Clinical Trial Registry: http://www.chictr.org.cn

# SYNOPSIS

**Background**

The global increase in the aging population is escalating challenges in healthcare and society, particularly with the prevalence of sarcopenia. Sarcopenia, a condition marked by loss of skeletal muscle mass and function is rising among the elderly, affecting 10-27% and severely increasing mortality risks. Current palliative interventions focus on exercise and nutritional supplementation, with no optimal regimen identified for sarcopenia. High-intensity interval training (HIIT) has gained traction for its potential benefits over traditional moderate-intensity continuous training (MICT), showing promising results in improving muscle mass and exercise capacity in older adults, particularly in those with post-cardiac intervention. Despite the advantages of HIIT in stimulating skeletal muscle and increasing protein synthesis, research comparing its effects to MICT on sarcopenia and the key sensors at play is scant.

**Aim of the trial:** This trial is to explore the differentially expressed plasma proteins induced by high-intensity interval training and moderate-intensity continuous training using omics technologies and bioinformatics methods.

**Type of the trial:** Randomized crossover trial.

**Participants**

**Inclusion criteria**

- Sedentary males aged 30-35 years;
- Generally in good health condition;
- Able to comply with and complete the exercise cardiopulmonary test evaluation;
- Willing and consents to participate in the study.
- Sedentary lifestyle in the past three months: sedentary is defined as individuals who exercise less than 3 hours per week and have more than 8 hours of static activity daily. Static activity time refers to the total time spent on activities such as using a computer, writing materials, attending meetings during work, and watching TV, playing computer games, playing chess, reading, etc., after work. Weekly exercise time refers to the total time the participant engages in fitness exercises of moderate intensity (slightly strenuous) or above each week.

**Exclusion criteria**

- Have musculoskeletal functional impairments that affect participation in exercise;
- Suffer from uncontrolled diseases;
- Have taken drugs that affect metabolism or cardiovascular response in the past two weeks;
- Have participated in systematic exercise training;
- Have dieted or attempted to lose weight in the past two weeks;
- Are currently participating in other clinical trials;
- Cannot confirm the ability to complete the study due to personal life arrangements, such as having plans for long-distance travel.

**Dropout criteria**

- The patient cannot tolerate the designated intensity of exercise training during the study;
- Serious safety issues arise during the trial;
- The patient develops new illnesses;
- There are significant changes in the patient's lifestyle and dietary habits during the trial;
- The researcher believes that further trials are not in the best interest of the participant;
- The patient revokes informed consent.

**Number of subjects:** N = 10

**Primary outcome:** Proteomic responses in plasma following MICT and HIIT

**Type of intervention** Exercise training

**Intervention protocols:**

**(1) MICT:** The MICT protocol consists of a 1-minute warm-up, followed by training at 50% maximum exercise capacity for 36 minutes, and concluding with a 4-minute cooldown, resulting in a total exercise duration of 41 minutes.

**(2) HIIT:** HIIT involves a 5-minute warm-up, followed by four repetitions of high-intensity training and a low-intensity recovery cycle, and ends with a 4-minute cooldown, also totaling 41 minutes. Each cycle of HIIT includes 4 minutes of high-intensity training at 70% maximum exercise capacity, followed by 4 minutes of low-intensity training at 30% maximum exercise capacity.

**Time plan:** Start: May 2023

First inclusion of participants: May 2023

Inclusion of all participants: Dependent

All participants completed the study: Dependent

**Statistical analysis:** The proteomic data will be analysed by an expert who is good at bioinformatics.

**
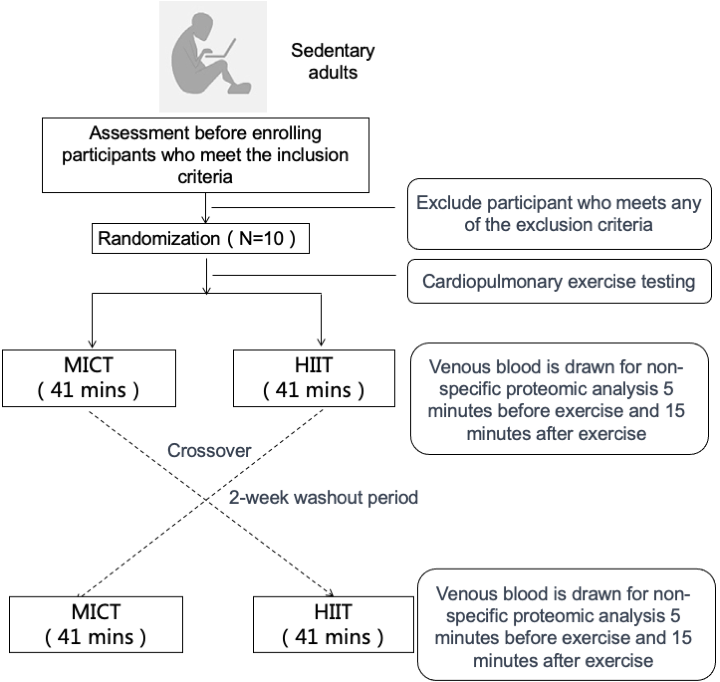
Flow chart**

**Evaluation and visit schedule**

**Table 1. Summary of examinations at different time-points**

| **Study Visits** | **MICT-first group** | **HIIT-first group** |
| --- | --- | --- |
| Screening (at the begining) | Medical history, physical activity habit assessment, anthropometric measurements. | |
| Visit 1 (2^nd^ week) | Cardiopulmonary exercise testing, resting ECG | |
| *Randomization* | | |
| Visit 2 (4^th^ week) | First time exercise: Venous blood will be drawn for non-specific proteomic analysis 5 minutes before exercise and 15 minutes after exercise. | |
| *Two weeks of washout period.* | | |
| Visit 3 (6^th^ week) | Second time exercise: Venous blood will be drawn for non-specific proteomic analysis 5 minutes before exercise and 15 minutes after exercise. | |

# INTRODUCTION

## 3.1 Rationale

A substantial body of research has shown that regular exercise can significantly improve the physiological functions of multiple organs and systems, reducing the incidence of cardiovascular diseases and the risk of all-cause mortality, and promoting heathy aging [1, 2]. Just like a drug prescription, an individualized exercise prescription is fundamental in promoting health benefits related to exercise and implementing precise treatments for participants. An exercise prescription mainly comprises factors like the mode of exercise, intensity, duration, and frequency [3]. Exercise intensity might be a key modulating factor, as the health benefits of 75 minutes of high-intensity exercise are similar to those produced by 150 minutes of moderate-intensity exercise per week [1, 4, 5]. Another research also indicates that HIIT for the same duration is more effective than MICT in improving metabolic syndrome and body composition in patients post-percutaneous coronary intervention [6, 7]. The differences in molecular responses induced by exercises of varying intensities are not yet clear. Exploring the molecular sensors and regulatory mechanisms of different exercise intensities can aid in advancing precise exercise therapy and provide a basis for the development of exercise mimetics.

Previous research on the mechanisms of exercise has largely centered on understanding how exercise affects specific molecules or pathways, constrained by the available research methodologies. Prior findings have demonstrated that exercise can positively influence mitochondrial quality control in both skeletal and cardiac muscles of mice. This is achieved through the activation of AMPK, which leads to improvements in insulin resistance and an enhancement in stress resistance. [8-12]. Recently, the development of multi-omics technologies and bioinformatics has provided methods to explore comprehensive exercise responses. Using these methods, researchers like Contrepois [13] and Whitham [14] have reported the acute molecular responses of healthy adults to a single session of moderate-intensity exercise. In their studies, during a single session of moderate-intensity exercise thousands of molecular changes and biological processes were revealed, including energy metabolism, oxidative stress, inflammatory responses, tissue repair, growth factor responses, and regulatory pathways. The comprehensive proteomic characteristics between different exercise intensities are not yet clear and have not been reported.

This study intends to use a crossover trial design, employing non-specific proteomics techniques and bioinformatics methods to map the molecular responses of adult plasma to MICT and HIIT. This research will be the first to explore proteins specifically and co-expressed in both forms of exercise, providing data support and direction for subsequent research in precise exercise therapy; it will also provide information for the development of exercise mimetics, allowing patients who are unable to participate in exercise due to functional impairments to also gain the health benefits associated with exercise.

**We hypothesize** that the HIIT will induce a distinctive plasma protein response in contrast to MICT.

## Objectives

**Primary Objective**

To delineate the proteomic responses in adult plasma following MICT and HIIT.

**Reference**

1.Visseren FLJ, Mach F, Smulders YM, Carballo D, Koskinas KC, Back M, et al. 2021 ESC Guidelines on cardiovascular disease prevention in clinical practice. Eur Heart J. 2021 Sep 7;42(34):3227-337.

2.Arnett DK, Blumenthal RS, Albert MA, Buroker AB, Goldberger ZD, Hahn EJ, et al. 2019 ACC/AHA Guideline on the Primary Prevention of Cardiovascular Disease: A Report of the American College of Cardiology/American Heart Association Task Force on Clinical Practice Guidelines. Circulation. 2019 Sep 10;140(11):e596-e646.

3.Fletcher GF, Ades PA, Kligfield P, Arena R, Balady GJ, Bittner VA, et al. Exercise standards for testing and training: a scientific statement from the American Heart Association. Circulation. 2013 Aug 20;128(8):873-934.

4.Piercy KL, Troiano RP, Ballard RM, Carlson SA, Fulton JE, Galuska DA, et al. The Physical Activity Guidelines for Americans. JAMA. 2018 Nov 20;320(19):2020-8.

5.Chinese Preventive Medicine Association, et al. Chinese Guideline on Healthy Lifestyle to Prevent Cardiometabolic Diseases. Chin Med J. 2020;35(3):1000-3614.

6.Dun Y, Thomas RJ, Medina-Inojosa JR, Squires RW, Huang H, Smith JR, et al. High-Intensity Interval Training in Cardiac Rehabilitation: Impact on Fat Mass in Patients With Myocardial Infarction. Mayo Clin Proc. 2019 Sep;94(9):1718-30.

7.Dun Y, Thomas RJ, Smith JR, Medina-Inojosa JR, Squires RW, Bonikowske AR, et al. High-intensity interval training improves metabolic syndrome and body composition in outpatient cardiac rehabilitation patients with myocardial infarction. Cardiovasc Diabetol. 2019 Aug 14;18(1):104.

8.Jiang L, Shen X, Dun Y, Xie M, Fu S, Zhang W, et al. Exercise combined with trimetazidine improves anti-fatal stress capacity through enhancing autophagy and heat shock protein 70 of myocardium in mice. Int J Med Sci. 2021;18(7):1680-6.

9.Li H, Dun Y, Zhang W, You B, Liu Y, Fu S, et al. Exercise improves lipid droplet metabolism disorder through activation of AMPK-mediated lipophagy in NAFLD. Life Sci. 2021 May 15;273:119314.

10.Xie M, Jiang L, Dun Y, Zhang W, Liu S. Trimetazidine combined with exercise improves exercise capacity and anti-fatal stress ability through enhancing mitochondrial quality control. Life Sci. 2019 May 1;224:157-68.

11.You B, Dun Y, Zhang W, Jiang L, Li H, Xie M, et al. Anti-insulin resistance effects of salidroside through mitochondrial quality control. J Endocrinol. 2020 Feb;244(2):383-93.

12.Dun Y, Liu S, Zhang W, Xie M, Qiu L. Exercise Combined with Rhodiola sacra Supplementation Improves Exercise Capacity and Ameliorates Exhaustive Exercise-Induced Muscle Damage through Enhancement of Mitochondrial Quality Control. Oxid Med Cell Longev. 2017;2017:8024857.

13.Contrepois K, Wu S, Moneghetti KJ, Hornburg D, Ahadi S, Tsai MS, et al. Molecular Choreography of Acute Exercise. Cell. 2020 May 28;181(5):1112-30.

14.Whitham M, Parker BL, Friedrichsen M, Hingst JR, Hjorth M, Hughes WE, et al. Extracellular Vesicles Provide a Means for Tissue Crosstalk during Exercise. Cell Metab. 2018 Jan 9;27(1):237-51.

# METHODS

## Trial design

This is an open-label, randomised crossover trial.

## Setting

Xiangya Hospital, Central South University, Changsha, China.

## Selection of trial participants

**Inclusion criteria**

Participants must meet ALL of the following criteria:

1. Sedentary males aged 30-35 years;
2. Generally in good health condition;
3. Able to comply with and complete the exercise cardiopulmonary test evaluation;
4. Willing and consents to participate in the study.
5. Sedentary lifestyle in the past three months: sedentary is defined as individuals who exercise less than 3 hours per week and have more than 8 hours of static activity daily. Static activity time refers to the total time spent on activities such as using a computer, writing materials, attending meetings during work, and watching TV, playing computer games, playing chess, reading, etc., after work. Weekly exercise time refers to the total time the participant engages in fitness exercises of moderate intensity (slightly strenuous) or above each week.

**Exclusion criteria**

Participants will be excluded for ANY ONE of the following reasons:

1. Have musculoskeletal functional impairments that affect participation in exercise;
2. Suffer from uncontrolled diseases;
3. Have taken drugs that affect metabolism or cardiovascular response in the past two weeks;
4. Have participated in systematic exercise training;
5. Have dieted or attempted to lose weight in the past two weeks;
6. Are currently participating in other clinical trials;
7. Cannot confirm the ability to complete the study due to personal life arrangements, such as having plans for long-distance travel.

**Dropout criteria**

1. The patient cannot tolerate the designated intensity of exercise training during the study;
2. Serious safety issues arise during the trial;
3. The patient develops new illnesses;
4. There are significant changes in the patient's lifestyle and dietary habits during the trial;
5. The researcher believes that further trials are not in the best interest of the participant;
6. The patient revokes informed consent.

**Premature termination of the trial**

Premature closure of a trial is to be considered if:

• There’s an insufficient recruitment rate

• The study deviates from and is not compliant with the protocol, or

• The quality of the data is insufficient.

The premature closure of a site will be decided by the coordinating investigator.

Investigators have to inform the coordinating investigator immediately when they decide not to take part in the trial any longer. The decision should be well-founded. Details on further treatment and follow-up of participants on the study have to be discussed with the coordinating investigator.

In case of the following situations, a premature termination of the trial has to be considered:

• Substantial changes in risk-benefit considerations

• New insights from other trials

• Insufficient efficacy stated in a futility analysis

• Insufficient recruitment rate

**Premature termination of trial participation**

Any individual premature termination during the training period and every premature termination of follow-up must be documented by the responsible investigator. The date, circumstances, and reason for the termination should be documented in detail.

## Sample size

This study is exploratory in nature, with the primary research indicator being the observation of any difference in plasma proteins or metabolic products after HIIT and MICT. Since there are currently no research reports comparing the proteomic or metabolomic results of HIIT and MICT, this study is based on the team's previously published research on HIIT and MICT improvements in adult fasting blood glucose, estimating a difference of 21.9 units for a certain protein or metabolic product between the two groups, with a standard deviation of 15.0 units (Yaoshan Dun, Randal J. Thomas, Joshua R. Smith et al. High-intensity interval training improves metabolic syndrome and body composition in outpatient cardiac rehabilitation patients with myocardial infarction. Cardiovascular Diabetology. 2019;18:104). With a Type I error α=0.05 and a test power of 1-*β*=0.8, the study design method selected is a 2*2 crossover trial design, with the outcome indicator type as a continuous variable. It was calculated that a total of 8 participants are needed, and considering a 20% dropout rate, 10 participants should ultimately be included. The calculations were completed using PASS 15.0.5 software.

## Interventions

**Training intervention**

**Type**: Bicycle.

**Setting**: Xiangya Hospital, Central South University, Changsha, China.

**Protocol:** A brief description of the exercise program is as follows:

- MICT: The MICT protocol consistes of a 1-minute warm-up, followed by training at 50% MEC for 36 minutes, and concluding with a 4-minute cooldown, resulting in a total exercise duration of 41 minutes.
- HIIT: The HIIT involves a 5-minute warm-up, followed by four repetitions of high-intensity training and a low-intensity recovery cycle, and ends with a 4-minute cooldown, also totaling 41 minutes. Each cycle of HIIT includes 4 minutes of high-intensity training at 70% MEC, followed by 4 minutes of low-intensity training at 30% MEC.

## Outcomes

**Primary outcome**

Any differentially expressed plasma proteins or metabolic molecules observed after HIIT and MICT.

**Secondary outcomes**

Differences in other plasma proteins or metabolic products, based on the completion of the primary outcome measures.

## Statistical methods

The Shapiro–Wilk test will be performed to determine normality for continuous variables. Descriptive statistics will be presented as mean ± SD for normally distributed continuous variables while those with a non-normal distribution as median interquartile range (IQR)..

In this study, proteomic data will be processed using Spectronaut Pulsar, with raw data files converted to a standard format by local normalization. The data will undergo pre-processing to remove low-quality spectra and to normalize signal intensities across samples. Peptide identification will be performed using the UniProt with a false discovery rate (FDR) of 0.01 to control for type I errors. Quantitative data analysis will be conducted using R, where peptide intensities will be compared across samples using *t*-test. Differential expression analysis will be performed to identify significantly regulated proteins, with a *P*-value < 0.05 and foldchange threshold of 1.2 considered statistically significant. Venn analysis will be applied to visualize shared and unique proteins between MICT and HIIT.

When comparing two group means, *t*-test will be used in an unpaired two-tailed fashion. For more than two groups, ANOVA with Bonferroni multiple comparisons test will be used. Adjusted Pearson correlation analysis will be used to assess the association between two factors. All analyses were performed using R (version 4.2.0) software. Statistical significance was set at *P*<0.05 (two-sided).

## Participant timeline

**Expected duration of the trial**

Duration per participant: 3 months.

**Time plan:** Start: May 2023

First inclusion of participants: May 2023

Inclusion of all participants: Dependent

All participants completed the study: Dependent

## Recruitment

All participants will be recruited from the Hospital.

## Assignment and allocation of intervention

After informed consent and screening, participants will be randomised (1:1) to the MICT-first group and HIIT-first groups.

**Sequence generation:** For allocation of participants, an investigator with no involvement in the intervention part of the trial will prepare a computer-generated list of random numbers that will be used to assign participants in a 1:1 ratio to the MICT-first and HIIT-first groups.

**Concealment mechanism:** The allocation sequence will be concealed from the researcher enrolling and assessing participants in sequentially numbered, opaque, sealed, and stapled envelopes.

**Implementation:** After acceptance of a participant by the panel, and before admission to a group, the appropriate numbered envelope will be opened; the card inside will inform if the participant is to be assigned to the MICT-first or the HIIT-first group, and this information will then be given to the researchers responsible for intervention.

## Blinding

Whereas patients and physicians allocated to the intervention group be aware of the allocated arm, outcome assessors and data analysts will be kept blinded to the allocation.

## Data management

**Anonymity and data security**

All participant information will be stored without their names or personal identification, or any other information which may reveal their identity. A special code will attach results and samples to a list of names. This list will only be accessible for selected authorised personnel performing this research.

**Access to source data**

According to the ICH-GCP and the applicable Chinese laws, the coordinating investigator must allow access to all authorised third parties to the trial site and insight into the source data. This permission includes the medical committee, clinical trial monitors, and authorised members of the Local Government.

**Monitoring**

Three regular visits will be scheduled. The reason for these visits is to check the compliance of the trial with the study protocol. This control includes a check of the informed consent documents of all participants, source data verification of the key data (eligibility criteria, components of the primary outcome, safety aspects) of all participants.

# INDIVIDUAL TRIAL PROCEDURE AND OUTCOMES ASSESSMENT

Table 2. Summary of the examinations

|  | T1 | T2 | T3 | | | T4 | T5 | | |
| --- | --- | --- | --- | --- | --- | --- | --- | --- | --- |
|  |  |  | T3A | T3B | T3C |  | T5A | T5B | T5C |
| Randomization | X |  |  |  |  |  |  |  |  |
| Demographics | X |  |  |  |  |  |  |  |  |
| Anthropometric assessment | X |  |  |  |  |  |  |  |  |
| Physical activity habit | X |  |  |  |  |  |  |  |  |
| Diet - food frequency questionnaire | X |  |  |  |  |  |  |  |  |
| Medical history | X |  |  |  |  |  |  |  |  |
| Medication history | X |  |  |  |  |  |  |  |  |
| ECG | X |  |  |  |  |  |  |  |  |
| Perceived Stress Scale | X |  |  |  |  |  |  |  |  |
| Generalized Anxiety Disorder Scale | X |  |  |  |  |  |  |  |  |
| Patient Health Questionnaire | X |  |  |  |  |  |  |  |  |
| Cardiopulmonary exercise testing | X |  |  |  |  |  |  |  |  |
| Whole blood collection, non-specific proteomics |  |  | X |  | X |  | X |  | X |
| Gas exchange parameters during exercise |  |  |  | X |  |  |  | X |  |
| Heart rate and blood pressures during exercise |  |  |  | X |  |  |  | X |  |

T1 = At enrollment (week 0); T2 = 2 weeks washout period after cardiopulmonary exercise testing (2^nd^ week); T3 = First exercise intervention (4^th^ week); T3A = 5 minutes before training; T3B = During HIIT/MICT training; T3C = 15 minutes after training; T4 = 2 weeks washout period for alternating between the two types of exercise; T5 = Second exercise intervention (6^th^ week); T5A = 5 minutes before training; T5B = During HIIT/MICT training; T5C = 15 minutes after training.

**Clinical assessment and exercise preparticipation screening**

The screening will take place following the initial enrolment and will take place in two visits. All participants will be screened by medical history and assessed with a physical examination and physical activity assessment in the initial visit. After the initial assessments, those who meet the inclusion criteria and meet the criteria for sedentary lifestyle will be screened in the second round of examinations. Examinations will be performed according to standard operating procedures and will include anthropometry, electrocardiogram, and cardiopulmonary exercise testing. The investigators conducting the evaluations will not be performing the randomisation at baseline testing.

**Demographics and physical activity assessment**

- Body weight, height, and BMI
- Physical activity: International Physical Activity Questionnaire-Short Form
- Diet - Food Frequency Questionnaire

**Clinical Data**

- Medical history
- Medication history
- Blood biochemical tests: fasting blood glucose (FBG), insulin sensitivity, complete blood count, glucose and lipid profiling, liver function and kidney function tests
- Resting electrocardiogram
- Stress level - Perceived Stress Scale
- Generalized Anxiety Disorder Scale (GAD-7)
- Patient Health Questionnaire (PHQ-9)

**Blood collection**

In this study, professional medical personnel will perform 4 venous blood collections for each participant, corresponding to before and after the two exercise interventions. Each blood collection will be 10 ml, collected using non-anticoagulant tubes, left at room temperature for 2 hours, then centrifuged at 2-8℃ at 1000g for 15 minutes, and the supernatant is taken and stored at -20℃ or -80℃ for subsequent testing and analysis.

**Proteomics procedures**

**Protein extraction**

Frozen samples (about 100 mg) will quickly be ground into fine and uniform powder in liquid nitrogen and then homogenized in 1 mL phenol extraction buffer, after which 1 mL saturated phenol with TrisHCl (pH 7.5) will be added. After several times’ shake, the mixture will be kept at 4℃ for 30 min. The upper phenolic phase will be separated from the aqueous phase by centrifugation at 7100 g at 4℃ for 10 min, transferred to a fresh tube and mixed with five volumes of pre-cold 0.1 M ammonium acetate-methanol. After being kept at −20℃ overnight, the mixture will be centrifuged at 12,000 g for 10 min at 4℃ to pellet precipitated protein. For wash step, the pellet will be resuspended twice with pre-cold methanol and twice with ice-cold acetone. Following another round of centrifugation, the pellet will be collected, air-dried and resuspended with 300 μL lysate solution. After incubation of 3 hr at room temperature, the solution will be centrifuged to remove any insoluble fraction and the resulting supernatant will contain the total extractable protein. The total protein concentrations will be quantified by bicinchoninic acid assay.

**Protein digestion**

According to the measured protein concentration, the same quality protein from each sample will be taken, and different groups of samples will be diluted to the same concentration and volume. Furthermore, 25 mM DTT of corresponding volume will be added into the above protein solution to make the DTT final concentration about 5 mM, and incubate at 55℃ for 30-60 min. Then the corresponding volume of iodoacetamide will be added so that the final concentration is about 10 mM, and place in the dark for 15-30 min at room temperature. Then 6 times the volume of precooled acetone in the above system to precipitate the protein, and place it at - 20°C for more than four hours or overnight. After precipitation, the sample will be taken out and centrifuged at 8000 g for 10 min at 4℃ for collecting the precipitate. According to the amount of protein, the corresponding volume of enzymolysis diluent (protein: enzyme = 50:1 (m/m), 100 μg of protein add 2 μg of enzyme) will be added to redissolve the protein precipitate, then the solutions will be incubated for digestion at 37 ℃ for 12 h. The samples will be desalted on SOLA™ SPE. After drying under vacuum, samples will be resuspended and iRT peptides (1:10) will be added.

**High-pH reversed-phase fractionation**

The peptide of pooled samples will be fractionated by 1100 HPLC System (Agilent). Mobile phases A (2% acetonitrile in HPLC water) and B (98% acetonitrile in HPLC water) will be used for RP gradient. The solvent gradient will be set as follows: 0~10 min, 98% A; 10~10.01 min, 98%95% A; 10.01~37 min, 95%80% A; 37~48 min, 8060% A; 48~48.01 min, 6010% A; 48.01~58 min, 10% A; 58~58.01 min, 1098% A; 58.01~63 min, 98% A. Tryptic peptides will be separated at a fluent flow rate of 250μL/min and monitored at 210 nm. Samples will be collected for 10-50 min, and eluent will be collected in centrifugal tube 1-10 every minute in turn. Samples will be recycled in this order until the end of gradient. The separated peptides will be lyophilized for mass spectrometry.

**Data dependent acquisition (DDA) and data independent acquisition (DIA) Mass spectrometry analysis**

The Proteomic data analysis will be performed by Shanghai Luming biological technology co., LTD (Shanghai, China). TimsTOF Pro mass spectrometer (Bruker) and Easy nanoLC-1200 (Thermo Fisher Scientific) will be used for both shotgun proteomics and DIA experiments. Samples will be loaded and separated by a C18 column (50 cm × 75 µm) on an EASY-nLCTM 1200 system (Thermo, USA). The flow rate will be 300 nL/min and linear gradient will be 90 min (0~45min, 5%B; 45~50 min, 27% B; 50~55 min, 100% B; 55~60 min, 100% B; mobile phase A = 0.1% FA in water and B = 0.1% FA in 80%ACN).

For data dependent acquisition (DDA), full scan MS spectra will be acquired at the resolution of 35,000, with the automatic gain control (AGC) target set as 1e6. The top 10 intense ions will be isolated for HCD MS/MS fragmentation. For data independent acquisition (DIA), a survey scan from 350 to 1250 m/z at 35,000 resolution will be adopted. Then, 26 DIA windows will be acquired and the stepped collision energy will be 28eV.

**Database search**

The default factory settings will be used for the Spectronaut Pulsar™ (Biognosys, Swiss) search and library generation (including Trypsyin/P as enzyme, up to two missed cleavages allowed, Oxidation of Me as variable modifications, carbamidomethyl of C as fixed modification, and 1% FDR for PSM, peptide and protein identification). The DDA search results will be imported into Spectronaut Pulsar™. The DIA data will be analysed with Spectronaut searching the above constructed spectral library. The main parameters of the software will be set as follows: retention time prediction type will be dynamic iRT, interference on MS2 level correction will be enabled, and cross run normalization will be enabled. All results will be filtered based on Q value cutoff 0.01 (equivalent to FDR < 1%).

**Cardiopulmonary exercise testing**

All participants will be tasked to perform a cardiopulmonary exercise testing with individually tailored ramp protocols on cycle ergometry on V2. The workload for the cardiopulmonary exercise testing will gradually be increased each minute by a workload calculated as the value of the predicted maximal work rate divided by 8 to 12 according to the individual’s health condition and activity habit so that the duration of cardiopulmonary exercise testing fits within 8-12 min.

A 12-lead ECG will be monitored continuously, and blood pressure will be measured by manual sphygmomanometry at rest and during the last 20-30 seconds of every two minutes. Oxygen saturation will also be monitored continuously via finger oximetry. Participants will be asked to score the Rating of Perceived Exertion accordingly (RPE, Borg 6-20) at the end of each stage.

Participants will be encouraged to continue exercising until they present with at least one of the following termination criteria:

1. achievement of ≥ 85% predicted maximal heart rate (predicted maximal heart rate = 220 - age);
2. plateau in heart rate with increasing workload;
3. plateau in oxygen consumption with increasing workload;
4. respiratory exchange ratio (RER) of ≥ 1.10;
5. rating of perceived exertion (RPE) ≥ 17 (Borg 6 - 20 scale);
6. angina increasing to 3 on a scale of 4;
7. severe fatigue or dyspnea;
8. a decrease in systolic blood pressure with increasing work rate;
9. severe supraventricular or ventricular arrhythmias;
10. signs of poor peripheral perfusion; or
11. the participant’s request to stop.

After test completion, a cool-down will be performed, with continued ECG monitoring, for 6 minutes or longer if the participant is symptomatic or if blood pressure, heart rate, and ST segments have not returned to near baseline values.

**Participant information and informed consent**

The investigator must explain the nature of the study to each trial participant, its purpose, the procedures involved, the expected duration, the potential risks, benefits involved, and any discomfort it may entail. Each trial participant must be informed that participation in the study is voluntary and that they may withdraw from the study at any time, and that withdrawal of consent will not affect their subsequent medical treatment or relationship with the treating physician. The participant should be provided with enough time to think about their participation in the study without any pressure.

The Informed Consent should be given by means of a standard written statement (please refer to the appendix). The trial subject should read the statement and consider their decision before signing and dating the document and should be given a copy of the signed document. No participant can enter the study before his/her Informed Consent has been obtained. No study-related procedures will be performed before Informed Consent is obtained. If written consent is for some reason not possible, oral consent can be obtained if witnessed by a signed statement from one or more persons not involved in the study, mentioning why the participant was unable to sign the form.

**Withdrawal of informed consent**

Participants may withdraw their consent to participate at any time of the trial without providing its reason. Nevertheless, the participant should be asked for the reason for the premature termination after being informed that they do not need to answer this question. The participant must be informed that choosing not to participate or to withdraw the consent will not affect their subsequent treatment or relationship with the study group. The date of enrolment and reason for withdrawal is to be documented in any case. The participant is to be informed that in case of revocation of their consent, the stored data may be used further, as may be necessary:

• to assess effects of the study intervention to be tested

• to guarantee that the interests of the participant are not impaired

• to comply with the regulatory requirements

**Enrolment and randomisation**

Randomization and allocation concealment: 1) Assign numbers to eligible participants who meet the inclusion criteria and do not meet any exclusion criteria; 2) Generate random numbers: An individual not involved in the intervention uses SPSS 26 software to sequentially generate 10 random numbers; 3) Number the participants in order and match them with the generated random numbers; 4) Group participants based on the matching results (the method is: sort the 10 random numbers from smallest to largest to obtain sequence numbers R, with R1–R5 designated for the group starting with MICT followed by HIIT, and R6–R10 for the group starting with HIIT followed by MICT), and place the group information into envelopes; 5) Give each newly enrolled participant the corresponding envelope containing group information in sequence, and directly enter the predetermined group to start the experiment. The above grouping operations are performed by individuals not involved in the intervention.

**Violation of eligibility criteria**

If for any reason, a participant who would have been included in the study turns out to have violated the eligibility criteria, investigators shall proceed as follows. In the case that this participant has not been randomised, immediately stop their participation in the entire trial. Since by this time, an ID code has been assigned to the participant, notify in the participant list that the participant has been withdrawn. Do not use this ID code for any other participant. If the participant has already been randomised, contact the lead investigators immediately. In general, the violation of eligibility criteria is not a reason for the premature withdrawal of the participant from the study. Stop the study participation immediately only in case the violated criteria represent an additional risk for the participant or in case the participant has been randomised by an error without his/her consent. In all other cases, proceed per the protocol and wait for instructions coming from the coordinating investigators.

**Adverse event (AE/SAE)**

**Adverse event (AE)**

An event that is an unfavourable or unintended sign, symptom, or disease associated with exercise training.

**Serious adverse event (SAE)**

Any long-term or temporary life-threatening condition associated with exercise training. These conditions mean any adverse experience that results in any of the following outcomes: death, inpatient hospitalisation (within 24 hours of last training session), persistent or significant disability/incapacity.

**Response to AEs**

In the case of an AE, the coordinating investigator MUST be notified within 24 hours, filing and submitting an SAE report via email to the investigators.

**Analysis of safety**

All AEs as part of the screening, baseline, or during the training period should be reported. In the context of this study, after baseline testing, AEs are those which could take place during the exercise intervention. Hence, it is mandatory to record and document all, which occur after the start and no later than two hours after the end of the exercise. The frequency of adverse events related to the exercise will be monitored. If required, the study protocol may be amended in terms of individual training dosage.

# ETHICAL AND LEGAL ASPECTS

## Ethical approval

This trial has been rigorously reviewed and approved by the Ethics Committee of Xiangya Hospital, Central South University, ensuring adherence to the ethical standards of the 1964 Declaration of Helsinki and its later amendments. This Randomized Crossover Trial (approval number 2023030379, registered at Chictr.org.cn under ChiCTR2300071797). The Involved participants will be asked to provided informed consent.

## Confidentiality

All participant information will be stored without their names or personal identification, or any other information which may reveal their identity. A special code will attach results and samples to a list of names.

## Others

**Participant information and informed consent**

Written and informed consent will be given, signed, and retrieved from all subjects at the beginning of the study, along with information sheets. The letter of informed consent will be provided to the participants in their native language (Chinese) and in terms understandable for the participants, followed by explanations from the investigators that should be clear to all participants. From there, participants will be afforded the right to:

- - To know that participation is voluntary
  - To withdraw themselves from the project at any time
  - To be given time to ask questions freely and receive answers before making a decision
  - To know of any benefits involved in participation
  - To know the degree of risk and burden involved in participation
  - To know the procedures that will be implemented in the case of incidental findings
  - To receive assurances that appropriate insurance cover is in place
  - To know how their data will be collected, protected during the project, and either destroyed or re-used at the end of the research; if plans to re-use the data exist, participants should be duly informed and also consented for this further usage
  - To know of any potential commercial exploitation of the research.

**Tests and associated risks**

If the participants fulfil the inclusion criteria and do not meet any condition that would exclude them from the study, we will perform several tests on each subject: cardiopulmonary exercise with cycle ergometry, assessments of measures of height, weight, waist:hip-ratio, in addition to health-related questionnaires. These tests will be performed before the training period. After testing, participants will be randomised to an exercise group or to a control group receiving modern advice.

Although the risk of an adverse event or serious adverse events is elevated during exercise compared to rest, this absolute increase of risk is low, and it is generally accepted that the benefits brought about by exercise outweigh the low-risk of adverse events. The cardiopulmonary exercise tests are aborted when the participant can no longer sustain or increase the exercise load, or by signs of ischemia and are undertaken with the supervision of clinicians. We recently published an article finding that cardiopulmonary exercise testing is a relatively safe form of testing in Chinese patients. The team has good experience in undertaking cardiopulmonary exercise testing and risk management.

**Advantages for participants**

In this project, each participant will receive a community-based circuit training program focused on improving different aspects of fitness, and hopefully improving cardiovascular health by decreasing several of the participant’s cardiovascular risk factors.

Regarding this research, there are no principal ethical concerns on physical exercise and conditioning, as these activities will improve cardiovascular and general health. Reduced exercise capacity and functionality as a consequence of inactivity is an important trigger of reduced quality of life, invalidity, and premature death. Therefore, any reduction of inactivity in adult population would be a benefit to general health.

**Adherence to the protocol**

This trial will be conducted in accordance with the laws of The People’s Republic of China and the ICH guidelines for Good Clinical Practice (GCP), taking into account the Declaration of Helsinki and all its revisions.

We define protocol violations as any deviations from the procedures outlined in this document, which may include:

• missed evaluations/ incorrect timing of evaluations

• non-compliance with the study intervention

It is the investigator's responsibility to make a reasonable effort to correct any protocol violation once the participant has been enrolled, in order to keep the subject in the study.

Major protocol violations must be immediately reported to the principal coordinating investigators during the study. All protocol violations will be listed and discussed in a roundtable led by the principal investigator and the nature of these violations will be defined.

Under the fast-paced working environment, however, it is possible that some minor variations could occur due to circumstances outside of the investigators' control. All such deviations will be documented in records, together with the reason for their occurrence, and where appropriate, detailed in the study report. The investigator makes every effort to record data according to the protocol.

**Dissemination Plan**

The results of this study will be disseminated to all participants who have taken part in the research via sharing the findings on the social media platforms.

# Informed Consent Form

**Title of research:** The Impact of High-Intensity Interval Training and Moderate-Intensity Continuous Training on Adult Plasma Proteomics

This research is designed to explore the differentially expressed plasma proteins induced by high-intensity interval training and moderate-intensity continuous training using omics technologies and bioinformatics methods.

As part of screening and follow-up, you will be asked to take part in some physical examinations. These tests include measurement of height and weight; a cardiopulmonary exercise testing. You will be asked to participate in a session of moderate-intensity continuous training, and a session of high-intensity interval training.

**Risks and Discomfort:** In this research, we ask participants to take a cardiopulmonary exercise testing. There exists the possibility of certain changes occurring during the test. These include abnormal blood pressure, fainting, irregular, fast, or slow heart rhythm, and in rare instances, heart attack, stroke, or death. Every effort will be made to minimise these occurrences during the test, and that emergency equipment and personnel are readily available to deal with these unusual situations should they occur. They incur small risks to injuries during exercise, including falls or muscle soreness.

**Benefits and compensation:** No financial compensation will be offered after participation in this study. The results obtained from the test will quantify your exercise capacity and may be helpful in evaluating what types of physical activities are appropriate and safe for you. Also, it is acknowledged that those participating in exercise could receive the benefit of increased cardiorespiratory fitness and improved flexibility, strength and balance.

**Confidentiality:** All personal information, including names, will be anonymised. The participant names or ID numbers will not be available to anyone. The results of the research will be published in a professional journal.

**Withdrawal:** Participation in this study is voluntary. You may refuse to participate without penalty. Each participant is free to withdraw their consent and end their participation at any time.

**Questions:** Any questions concerning this research or participation in this project may be directed to the following email, dunyaoshan@csu.edu.cn.

**Agreement**

This agreement states that you have received a copy of this informed consent. Your signature below indicates that you agree to participate in this study.

Signature of participant ________________ Date _________

Subject name _______________

Signature of researcher ________________

# CONSORT Checklist

|  |  | Reporting Item | Page Number |
| --- | --- | --- | --- |
| **Title and Abstract** |  |  |  |
| Title | #1a | Identification as a randomized trial in the title. | 1 |
| Abstract | #1b | Structured summary of trial design, methods, results, and conclusions | 2-4 |
| **Introduction** |  |  |  |
| Background and objectives | #2a | Scientific background and explanation of rationale | 5-6 |
| Background and objectives | #2b | Specific objectives or hypothesis | 6 |
| **Methods** |  |  |  |
| Trial design | #3a | Description of trial design (such as parallel, factorial) including allocation ratio. | 7 |
| Trial design | #3b | Important changes to methods after trial commencement (such as eligibility criteria), with reasons | NA |
| Participants | #4a | Eligibility criteria for participants | sDocument 1. 12-14 |
| Participants | #4b | Settings and locations where the data were collected | Cover letter |
| Interventions | #5 | The experimental and control interventions for each group with sufficient details to allow replication, including how and when they were actually administered | sDocument 1. 15-16 |
| Outcomes | #6a | Completely defined prespecified primary and secondary outcome measures, including how and when they were assessed | sDocument 1. 16, 19 |
| Outcomes | #6b | Any changes to trial outcomes after the trial commenced, with reasons | NA |
| Sample size | #7a | How sample size was determined. | sDocument 1. 14–15 |
| Sample size | #7b | When applicable, explanation of any interim analyses and stopping guidelines | NA |
| Randomization - Sequence generation | #8a | Method used to generate the random allocation sequence. | sDocument 1. 17–18 |
|  |  |  |  |
| Randomization - Sequence generation | #8b | Type of randomization; details of any restriction (such as blocking and block size) | sDocument 1. 17–18 |
|  |  |  |  |
| Randomization - Allocation concealment mechanism | #9 | Mechanism used to implement the random allocation sequence (such as sequentially numbered containers), describing any steps taken to conceal the sequence until interventions were assigned | sDocument 1. 17–18 |
| Randomization - Implementation | #10 | Who generated the allocation sequence, who enrolled participants, and who assigned participants to interventions | sDocument 1. 17–18 |
| Blinding | #11a | If done, who was blinded after assignment to interventions (for example, participants, care providers, those assessing outcomes) and how. | sDocument 1. 17–18 |
| Blinding | #11b | If relevant, description of the similarity of interventions | sDocument 1. 17–18 |
| Statistical methods | #12a | Statistical methods used to compare groups for primary and secondary outcomes | 11–12 |
| Statistical methods | #12b | Methods for additional analyses, such as subgroup analyses and adjusted analyses | 11–12 |
| **Results** |  |  |  |
| Participant flow diagram (strongly recommended) | #13a | For each group, the numbers of participants who were randomly assigned, received intended treatment, and were analysed for the primary outcome | 12 |
| Participant flow | #13b | For each group, losses and exclusions after randomization, together with reason | sDocument 4. 8 |
| Recruitment | #14a | Dates defining the periods of recruitment and follow-up | 7 |
| Recruitment | #14b | Why the trial ended or was stopped | NA |
| Baseline data | #15 | A table showing baseline demographic and clinical characteristics for each group | sDocument 4.  2–3 |
| Numbers analysed | #16 | For each group, number of participants (denominator) included in each analysis and whether the analysis was by original assigned groups | sDocument 4.  8 |
| Outcomes and estimation | #17a | For each primary and secondary outcome, results for each group, and the estimated effect size and its precision (such as 95% confidence interval) | 12–14 |
| Outcomes and estimation | #17b | For binary outcomes, presentation of both absolute and relative effect sizes is recommended | NA |
| Ancillary analyses | #18 | Results of any other analyses performed, including subgroup analyses and adjusted analyses, distinguishing pre-specified from exploratory | 12–14 |
| Harms | #19 | All important harms or unintended effects in each group (For specific guidance see CONSORT for harms) | NA |
| **Discussion** |  |  |  |
| Limitations | #20 | Trial limitations, addressing sources of potential bias, imprecision, and, if relevant, multiplicity of analyses | 22 |
| Generalisability | #21 | Generalisability (external validity, applicability) of the trial findings | 19 |
| Interpretation | #22 | Interpretation consistent with results, balancing benefits and harms, and considering other relevant evidence | 20–22 |
| Registration | #23 | Registration number and name of trial registry | Cover letter |
| **Other information** |  |  |  |
| Interpretation | #22 | Interpretation consistent with results, balancing benefits and harms, and considering other relevant evidence | NA |
| Registration | #23 | Registration number and name of trial registry | Cover letter |
| Protocol | #24 | Where the full trial protocol can be accessed, if available | sDocument 1. |
| Funding | #25 | Sources of funding and other support (such as supply of drugs), role of funders | Cover letter |
