# Supplementary material for: High‐Intensity Interval Training Mitigates Sarcopenia and Suppresses the Myoblast Senescence Regulator EEF1E1
Source: J Cachexia Sarcopenia Muscle. 2024 Sep 14;15(6):2574–85. doi: 10.1002/jcsm.13600 (PMC11634493; doi:10.1002/jcsm.13600)
Supplement: Supplementary file 2 — Data S2 Supporting Information [file JCSM-15-2574-s004.docx]

**Supplementary document 2: Sarcopenia case-control study protocol**

Contents

[1. SYNOPSIS 2](#_Toc157704704)

[2. INTRODUCTION 5](#_Toc157704705)

[2.1 Rationale 5](#_Toc157704706)

[2.2 Objectives 5](#_Toc157704707)

[3. METHODS 5](#_Toc157704708)

[3.1 Study design 5](#_Toc157704709)

[3.2 Setting 6](#_Toc157704710)

[3.3 Selection of participants 6](#_Toc157704711)

[3.4 Sample size 7](#_Toc157704712)

[3.5 Outcomes 8](#_Toc157704713)

[3.6 Statistical methods 9](#_Toc157704714)

[3.7 Participant timeline 9](#_Toc157704715)

[3.8 Recruitment 9](#_Toc157704716)

[3.9 Data management 9](#_Toc157704717)

[4. OUTCOMES ASSESSMENT 10](#_Toc157704718)

[5. ETHICAL AND LEGAL ASPECTS 14](#_Toc157704719)

[5.1 Ethical approval 14](#_Toc157704720)

[5.2 Confidentiality 15](#_Toc157704721)

[5.3 Others 15](#_Toc157704722)

[6. Informed Consent Form 17](#_Toc157704723)

[7. STROBE Checklist 19](#_Toc157704724)

# SYNOPSIS

**Background**

Sarcopenia, also known as muscle wasting disease, is a condition associated with aging that leads to a decline in skeletal muscle mass, strength, and/or function. It results in increased clinical adverse events such as falls and rehospitalization, thereby becoming one of the leading causes of disability and death in the elderly. Sarcopenia is also related to a variety of chronic diseases such as cardiovascular disease, metabolic syndrome, and frailty, significantly affecting the quality of life of the elderly. However, sarcopenia is a disease with low awareness and high prevalence among the elderly, invisibly harming the health of older adults and reducing their quality of life, bringing a heavy burden to individuals, society, and the economy. Therefore, sarcopenia has become a hot topic of research in the field of geriatric medicine globally.

The possibility of preventing, delaying, treating, and even reversing sarcopenia through early and effective intervention measures is increasingly recognized. Current interventions for sarcopenia mainly include exercise, nutritional, and pharmacological interventions. However the mechanism underlying exercise therapy improves sarcopenia remain unclear.

The research team has previously studied differentially expressed proteins in healthy adults after MICT and HIIT and found that the HIIT-specifically expressed protein eukaryotic translation elongation factor 1 epsilon 1 (EEF1E1) may be related to sarcopenia. This study intends to verify the relationship between EEF1E1 and sarcopenia in elderly patients with the condition, providing a target for subsequent mechanistic exploration.

**Aim of the study**: this study is to investigate the association between EEF1E1 and sarcopenia.

**Type of the study**: Case-control study.

**Participants**

**Inclusion criteria**

Older adults aged 60-85;

Voluntarily participate in this study and sign an informed consent form.

**Exclusion criteria**

Those with obvious disabilities, requiring assistance for walking, unable to participate in grip strength and walking speed tests;

Those with serious diseases that limit exercise testing and training (such as Parkinson's disease, motor neuron disease, post-stroke sequelae, etc.) affecting functional activity;

Those with major organ failure;

Those with malignant tumor;

Those with a severe history of mental illness.

**Dropout criteria**

Serious safety issues arise during the study;

The patient revokes informed consent.

**Number of subjects:** N = 84

**Intervention**: NA

**Primary outcome:** The association between EEF1E1 and sarcopenia.

**Secondary outcomes:** The associations between EEF1E1 and skeletal muscle index and strength.

**Time plan:**

Start: September 2023

First inclusion of participants: September 2023

Inclusion of all participants: Dependent

All participants completed the study: Dependent

**Statistical analysis：** The Shapiro–Wilk test will be performed to determine normality for continuous variables. Descriptive statistics will be presented as mean ± SD for normally distributed continuous variables while as median interquartile range (IQR) for those with a non-normal distribution. When comparing two group means, *t*-test will be used in an unpaired two-tailed fashion. Adjusted Pearson correlation analysis will be used to assess the association between two factors. All analyses will be performed using R (version 4.2.0) software. Statistical significance will be set at *P*<0.05 (two-sided).

# INTRODUCTION

## 2.1 Rationale

Sarcopenia, a muscle-wasting disease associated with aging, leads to a decrease in muscle mass, strength, and function, potentially causing falls, rehospitalizations, disability, and even death among the elderly. Sarcopenia also correlates with chronic diseases like cardiovascular disease and metabolic syndrome, affecting the elderly's quality of life. Despite its prevalence and impact, sarcopenia remains underrecognized. Addressing this, our research team will investigate the association between the protein EEF1E1 that's differentially expressed following HIIT over MICT. The study aims to explore the connection between EEF1E1 and sarcopenia, which may serve as a focal point for further research into treatment mechanisms.

**We hypothesize** that the plasma EEF1E1 is significantly associated with the presence of sarcopenia.

## Objectives

**Primary Objective**

To explore the correlation between plasma EEF1E1 and sarcopenia in the elderly.

**Secondary Objectives**

To investigate the relationship between plasma EEF1E1 and skeletal muscle mass and strength.

# METHODS

## Study design

This is a cross-sectional study.

## Setting

Xiangya Hospital, Central South University, Changsha, China.

## Selection of participants

**Inclusion criteria**

Participants must meet ALL of the following criteria:

1. Older adults aged 60-85;
2. Voluntarily participate in this study and sign an informed consent form.

**Exclusion criteria**

Participants will be excluded for ANY ONE of the following reasons:

1. Those with obvious disabilities, requiring assistance for walking, unable to participate in grip strength and walking speed tests;
2. Those with serious diseases that limit exercise testing and training (such as Parkinson's disease, motor neuron disease, post-stroke sequelae, etc.) affecting functional activity;
3. Those with major organ failure;
4. Those with malignant tumors;
5. Those with a severe history of mental illness.

**Dropout criteria**

1. Serious safety issues arise during the study;
2. The patient revokes informed consent.

**Premature termination of the study**

Premature closure of a study is to be considered if:

• There’s an insufficient recruitment rate

• The study deviates from and is not compliant with the protocol, or

• The quality of the data is insufficient.

The premature closure of a site will be decided by the coordinating investigator.

Investigators have to inform the coordinating investigator immediately when they decide not to take part in the study any longer. The decision should be well-founded. Details on further treatment and follow-up of participants on the study have to be discussed with the coordinating investigator.

In case of the following situations, a premature termination of the study has to be considered:

• Substantial changes in risk-benefit considerations

• New insights from other studys

• Insufficient efficacy stated in a futility analysis

• Insufficient recruitment rate

**Premature termination of study participation**

Any individual premature termination during the training period and every premature termination of follow-up must be documented by the responsible investigator. The date, circumstances, and reason for the termination should be documented in detail.

## Sample size

This study is designed as a case-control study. Currently, there are no studies on the levels of exercise-induced differentially expressed EEF1E1 in the population with sarcopenia. Therefore, IGF-1 is used as the main indicator. Referring to previous literature (Yee ML, Hau R, Taylor A, Guerra M, Guerra P, Darzins P, Gilfillan C. Sarcopenia in women with hip fracture: A comparison of hormonal biomarkers and their relationship to skeletal muscle mass and function. *Osteoporos Sarcopenia*. 2020 Sep;6(3):139-145), the IGF-1 level in the sarcopenia group is 12.09±5.14, and in the non-sarcopenia group, it is 15.89±6.26, with α=0.05, power=90%, and bilateral testing, the calculated n=33. Considering a 25% dropout rate, the number in the sarcopenia group N=42. Ultimately, the case group needs at least 42 subjects for the study, and the control group 42 subjects.

**Match matching criteria and the number of controls per case**

**Matching Criteria:**

- Age: Cases will be matched with controls within a 2-year age range to ensure that age-related factors do not confound the results.

- Biologcial sex: Cases will be matched with controls based on biological sex to account for any gender-specific risk factors or differences in disease prevalence.

**Control-to-Case Ratio:**

- 1:1 ratio: Each case will be matched with one control of the same biological sex and within a 2-year age range.

## Outcomes

**Primary outcome**

The association between EEF1E1 and sarcopenia.

**Secondary outcomes**

The associations between EEF1E1 and skeletal muscle index and strength.

## Statistical methods

The Shapiro–Wilk test will be performed to assess normality for continuous variables. Descriptive statistics will be presented as mean ± SD for normally distributed continuous variables and as median with interquartile range (IQR) for those with a non-normal distribution.

When comparing means between two groups, a two-tailed unpaired *t*-test will be used. Adjusted Pearson correlation analysis will be used to assess the association between two factors. All analyses will be conducted using R (version 4.2.0) software. Statistical significance will be set at *P*<0.05 (two-sided).

## Participant timeline

**Expected duration of the study**

Duration per participant: two months.

**Time plan:** Start: September 2023

First inclusion of participants: September 2023

Inclusion of all participants: Dependent

All participants completed the study: Dependent

## Recruitment

All participants will be recruited from the community.

## Data management

**Anonymity and data security**

All participant information will be stored without their names or personal identification, or any other information which may reveal their identity. A special code will attach results and samples to a list of names. This list will only be accessible for selected authorised personnel performing this research.

**Access to source data**

According to the ICH-GCP and the applicable Chinese laws, the coordinating investigator must allow access to all authorised third parties to the study site and insight into the source data. This permission includes the medical committee, clinical study monitors, and authorised members of the Local Government.

# OUTCOMES ASSESSMENT

**Demographic characteristics**

- birth gender
- age
- educational level
- occupation
- anthropometric parameters (weight, height, body mass index, waist circumference, hip circumference, waist-to-hip ratio, upper arm circumference, calf circumference).

**Personal history and lifestyle habits**

- Information on smoking history, drinking history, history of sedentary lifestyle, and exercise habits;
- Physical activity level (using the Duke Activity Status Index, DASI), nutritional status (using the Mini Nutritional Assessment Short-Form, MNA-SF).

**Medical history**: hypertension, diabetes, cerebrovascular disease, tumors, hyperlipidemia, etc.

**Medication history**: drugs for thyroid function, hormonal drugs, long-term use of steroids and weight loss drugs, etc.

**Muscle strength:** Handgrip strength test: the participant holds a grip strength meter with their dominant hand, arm hanging naturally at the side of the body, then grips the meter as hard as possible while keeping the arm straight. The test is repeated twice, and the highest reading of the grip strength meter is taken.

**Body composition**: will be detected using the MC-780MA bioelectrical impedance analysis body composition analyzer, including indicators such as limb muscle mass.

**Physical performance**

- 30-second chair stand test: the participant sits on a chair with their back against the wall, hands crossed naturally at the chest, then stands up completely (knees straight) without assistance, and sits back down (buttocks fully touching the chair) for one count. The maximum number of times this can be done in 30 seconds is recorded.
- 6-meter walk speed: the distance of 6 meters is measured accurately, and the participant walks at their normal pace, with the time recorded by a stopwatch (accurate to 0.01s). The test is repeated twice and the average value is taken.
- 5-times sit-to-stand time: similar to the 30-second chair stand test, but the time taken to complete 5 sit-to-stand movements is recorded.

**Laboratory examination:** routine blood test, liver function, kidney function, blood sugar, blood lipids, EEF1E1, blood pressure, electrocardiogram, etc.


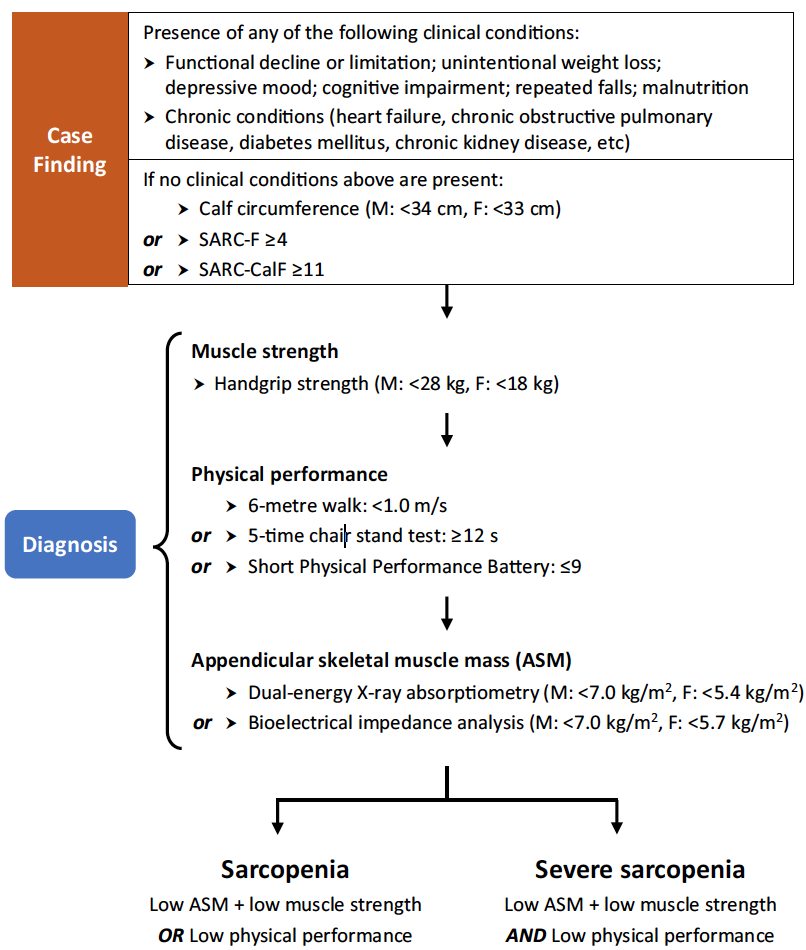
**Criteria of sarcopenia**: the screening will be initially conducted using calf circumference, followed by comprehensive assessment of limb skeletal muscle content, muscle strength, and physical function. The procedures for sarcopenia diagnosis will follow the criteria proposed by the 2019 AWGS include.

**Participant information and informed consent**

The investigator must explain the nature of the study to each study participant, its purpose, the procedures involved, the expected duration, the potential risks, benefits involved, and any discomfort it may entail. Each study participant must be informed that participation in the study is voluntary and that they may withdraw from the study at any time, and that withdrawal of consent will not affect their subsequent medical treatment or relationship with the treating physician. The participant should be provided with enough time to think about their participation in the study without any pressure.

The Informed Consent should be given by means of a standard written statement (please refer to the appendix). The study subject should read the statement and consider their decision before signing and dating the document and should be given a copy of the signed document. No participant can enter the study before his/her Informed Consent has been obtained. No study-related procedures will be performed before Informed Consent is obtained. If written consent is for some reason not possible, oral consent can be obtained if witnessed by a signed statement from one or more persons not involved in the study, mentioning why the participant was unable to sign the form.

**Withdrawal of informed consent**

Participants may withdraw their consent to participate at any time during the study without providing a reason. However, participants may be asked for their reason for withdrawing, although they are not obligated to provide an answer.The participant must be informed that choosing not to participate or to withdraw the consent will not affect their subsequent treatment or relationship with the study group. The date of enrolment and date of and reason for withdrawal is to be documented in any case. The participant is to be informed that in case of revocation of their consent, the stored data may be used further, as may be necessary:

• to guarantee that the interests of the participant are not impaired

• to comply with the regulatory requirements

**Violation of eligibility criteria**

If for any reason, a participant who would have been included in the study turns out to have violated the eligibility criteria, investigators shall proceed as follows. In the case that this participant has not been randomised, immediately stop their participation in the entire study. Since by this time, an ID code has been assigned to the participant, notify in the participant list that the participant has been withdrawn. Do not use this ID code for any other participant. If the participant has already been randomised, contact the lead investigators immediately. In general, the violation of eligibility criteria is not a reason for the premature withdrawal of the participant from the study. Stop the study participation immediately only in case the violated criteria represent an additional risk for the participant or in case the participant has been randomised by an error without his/her consent. In all other cases, proceed per the protocol and wait for instructions coming from the coordinating investigators.

**Adverse event (AE)**

No intervention will be conducted. Thus, the risk of AE is low. In the case of an AE, the coordinating investigator MUST be notified within 24 hours, filing and submitting an SAE report via email to the office email.

# ETHICAL AND LEGAL ASPECTS

## Ethical approval

The study was reviewed and approved by the Ethics Committee.

## Confidentiality

All participant information will be stored without their names or personal identification, or any other information which may reveal their identity. A special code will attach results and samples to a list of names.

## Others

**Participant information and informed consent**

Written and informed consent will be given, signed, and retrieved from all subjects at the beginning of the study, along with information sheets. The letter of informed consent will be provided to the participants in their native language (Chinese) and in terms understandable for the participants, followed by explanations from the investigators that should be clear to all participants. From there, participants will be afforded the right to:

- - To know that participation is voluntary
  - To withdraw themselves from the project at any time
  - To be given time to ask questions freely and receive answers before making a decision
  - To know of any benefits involved in participation
  - To know the degree of risk and burden involved in participation
  - To know the procedures that will be implemented in the case of incidental findings
  - To receive assurances that appropriate insurance cover is in place
  - To know how their data will be collected, protected during the project, and either destroyed or re-used at the end of the research; if plans to re-use the data exist, participants should be duly informed and also consented for this further usage
  - To know of any potential commercial exploitation of the research.

**Advantages for participants**

In this project, each participant will receive a community-based circuit training program focused on improving different aspects of fitness, and hopefully preventing or improving sarcopenia.

**Adherence to the protocol**

This study will be conducted in accordance with the laws of The People’s Republic of China and the ICH guidelines for Good Clinical Practice (GCP), taking into account the Declaration of Helsinki and all its revisions.

We define protocol violations as any deviations from the procedures outlined in this document, which may include:

• missed evaluations/ incorrect timing of evaluations

• non-compliance with the study intervention

It is the investigator's responsibility to make a reasonable effort to correct any protocol violation once the participant has been enrolled, in order to keep the subject in the study.

Major protocol violations must be immediately reported to the principal coordinating investigators during the study. All protocol violations will be listed and discussed in a roundtable led by the principal investigator and the nature of these violations will be defined.

Under the fast-paced working environment, however, it is possible that some minor variations could occur due to circumstances outside of the investigators' control. All such deviations will be documented in records, together with the reason for their occurrence, and where appropriate, detailed in the study report. The investigator makes every effort to record data according to the protocol.

**Dissemination Plan**

The results of this study will be disseminated to all participants who have taken part in the research via sharing the findings on the social media platforms.

# Informed Consent Form

**Title of research:** Preliminary Exploration of the Relationship between Exercise-Induced EEF1E1 and Sarcopenia in the Older Adults

This research is designed to explore the association between the differentially expressed plasma proteins induced by high-intensity interval training, EEF1E1, and sarcopenia.

As part of screening and follow-up, you will be asked to take part in some physical examinations, questionnaires, and medical tests. These tests include measurement of height and weight; a sarcopenia screening, routine blood tests.

**Risks and Discomfort:** In this research, we ask participants to take blood tests. You may feel little uncomfortable during blood collection. Every effort will be made to minimise this occurrence during the test, and that emergency equipment and personnel are readily available to deal with these unusual situations should they occur.

**Benefits and compensation:** No financial compensation will be offered after participation in this study. The results obtained from the test will quantify your status of sarcopenia and may be helpful in evaluating what types of physical activities are appropriate and safe for you.

**Confidentiality:** All personal information, including names, will be anonymised. The participant names or ID numbers will not be available to anyone. The results of the research will be published in a professional journal.

**Withdrawal:** Participation in this study is voluntary. You may refuse to participate without penalty. Each participant is free to withdraw their consent and end their participation at any time.

**Agreement**

This agreement states that you have received a copy of this informed consent. Your signature below indicates that you agree to participate in this study.

Signature of participant ________________ Date _________

Subject name _______________

Signature of researcher ________________

# STROBE Checklist

**Checklist of items that should be included in reports of case-control studies**

|  | Item No | Recommendation | Page No |
| --- | --- | --- | --- |
| **Title and abstract** | 1 | (*a*) Indicate the study’s design with a commonly used term in the title or the abstract | 1 |
|  |  | (*b*) Provide in the abstract an informative and balanced summary of what was done and what was found | 2-3 |
| Introduction | | | |
| Background/rationale | 2 | Explain the scientific background and rationale for the investigation being reported | 5-6 |
| Objectives | 3 | State specific objectives, including any prespecified hypotheses | 6 |
| Methods | | | |
| Study design | 4 | Present key elements of study design early in the paper | 8 |
| Setting | 5 | Describe the setting, locations, and relevant dates, including periods of recruitment, exposure, follow-up, and data collection | Cover letter |
| Participants | 6 | (*a*) Give the eligibility criteria, and the sources and methods of case ascertainment and control selection. Give the rationale for the choice of cases and controls | sDocument 2. 6 |
|  |  | (*b*) For matched studies, give matching criteria and the number of controls per case | sDocument 2. 8 |
| Variables | 7 | Clearly define all outcomes, exposures, predictors, potential confounders, and effect modifiers. Give diagnostic criteria, if applicable | sDocument 2. 10-12 |
| Data sources/ measurement | 8* | For each variable of interest, give sources of data and details of methods of assessment (measurement). Describe comparability of assessment methods if there is more than one group | sDocument 2. 10-12 |
| Bias | 9 | Describe any efforts to address potential sources of bias | 22 |
| Study size | 10 | Explain how the study size was arrived at | sDocument 2. 7 |
| Quantitative variables | 11 | Explain how quantitative variables were handled in the analyses. If applicable, describe which groupings were chosen and why | sDocument 2. 9 |
| Statistical methods | 12 | (*a*) Describe all statistical methods, including those used to control for confounding | sDocument 2. 9 |
|  |  | (*b*) Describe any methods used to examine subgroups and interactions | sDocument 2. 9 |
|  |  | (*c*) Explain how missing data were addressed | NA |
|  |  | (*d*) If applicable, explain how matching of cases and controls was addressed | sDocument 2. 8 |
|  |  | (*e*) Describe any sensitivity analyses | sDocument 2. 9 |
| Results | | | |
| Participants | 13* | (a) Report numbers of individuals at each stage of study—eg numbers potentially eligible, examined for eligibility, confirmed eligible, included in the study, completing follow-up, and analysed | NA |
|  |  | (b) Give reasons for non-participation at each stage | NA |
|  |  | (c) Consider use of a flow diagram | NA |
| Descriptive data | 14* | (a) Give characteristics of study participants (eg demographic, clinical, social) and information on exposures and potential confounders | sDocument 4. 5 |
|  |  | (b) Indicate number of participants with missing data for each variable of interest | NA |
| Outcome data | 15* | Report numbers in each exposure category, or summary measures of exposure | sDocument 4. 5 |

| Main results | | 16 | (*a*) Give unadjusted estimates and, if applicable, confounder-adjusted estimates and their precision (eg, 95% confidence interval). Make clear which confounders were adjusted for and why they were included | 13-14 |
| --- | --- | --- | --- | --- |
|  |  |  | (*b*) Report category boundaries when continuous variables were categorized | 13-14 |
|  |  |  | (*c*) If relevant, consider translating estimates of relative risk into absolute risk for a meaningful time period | 27 |
| Other analyses | 17 | Report other analyses done—eg analyses of subgroups and interactions, and sensitivity analyses | | NA |
| Discussion | | | | |
| Key results | 18 | Summarise key results with reference to study objectives | | 19 |
| Limitations | 19 | Discuss limitations of the study, taking into account sources of potential bias or imprecision. Discuss both direction and magnitude of any potential bias | | 22 |
| Interpretation | 20 | Give a cautious overall interpretation of results considering objectives, limitations, multiplicity of analyses, results from similar studies, and other relevant evidence | | 19-22 |
| Generalisability | 21 | Discuss the generalisability (external validity) of the study results | | 19 |
| Other information | | | | |
| Funding | 22 | Give the source of funding and the role of the funders for the present study and, if applicable, for the original study on which the present article is based | | Cover letter |

*Give information separately for cases and controls.
