# Supplementary material for: High‐Intensity Interval Training Mitigates Sarcopenia and Suppresses the Myoblast Senescence Regulator EEF1E1
Source: J Cachexia Sarcopenia Muscle. 2024 Sep 14;15(6):2574–85. doi: 10.1002/jcsm.13600 (PMC11634493; doi:10.1002/jcsm.13600)
Supplement: Supplementary file 3 — Data S3 Supporting Information [file JCSM-15-2574-s001.docx]

**Supplementary document 3**

**Animal and in vitro experimental methods**

Contents

[Animals study design 2](#_Toc153034565)

[Sample size calculation and statistical power 2](#_Toc153034566)

[Animal purchase and treatment 2](#_Toc153034567)

[Exercise training program 3](#_Toc153034568)

[Maximal running test 4](#_Toc153034569)

[Forelimb grip strength test 4](#_Toc153034570)

[Hanging grid test 5](#_Toc153034571)

[Tissue processing 5](#_Toc153034572)

[Wheat germ agglutinin (WGA) staining 5](#_Toc153034573)

[Immunofluorescence staining of paraffin section 6](#_Toc153034574)

[Transmission electron microscope (TEM) 6](#_Toc153034575)

[Cell experiments design 7](#_Toc153034576)

[Sample size (repetitions) calculation and statistical power 7](#_Toc153034577)

[Cell treatments 7](#_Toc153034578)

[siRNA transfection 8](#_Toc153034579)

[SA-β-gal staining 9](#_Toc153034580)

[Myotube differentiation 9](#_Toc153034581)

[Cell cycle assessment 9](#_Toc153034582)

[Cell immunofluorescence staining 10](#_Toc153034583)

[Western blot procedures 10](#_Toc153034584)

[RT-PCR procedures 11](#_Toc153034585)

[Statistical analysis 12](#_Toc153034586)

[ARRIVE checklist 13](#_Toc153034587)

# Animals study design

## Sample size calculation and statistical power

PASS software was used to calculate sample size of animal experiment. The number of mice for our ANOVA study was calculated using standard power calculations with α = 0.05 and a power of 0.8. Based on previous studies [1,2], we estimated the grip strength mean difference would be 100%, 115%, and 130% with the standard deviation of 4% in A-sed, A-MICT, and A-HIIT groups respectively, and estimated the mean differences is 100% and 43% with the standard deviation of 5% in Y-Sed and A-sed groups. As a result, 6.81 mice per group were needed. Considering possible exclusions, we set 8 mice per group and 32 mice for four groups in total.

## Animal purchase and treatment

All procedures with mice followed the guidelines for the use of live animals and were approved by the Medicine Animal Welfare Committee. This study was conducted under the ARRIVE guidelines.

3-month-old and 20-month-old male C57BL/6J mice were purchased from the Laboratory Animal Centre. The 3-month-old mice were used as young control groups and subjected to a sedentary condition (Young). The 20-month-old aged mice were numbered according to body weight, and the randomization function in SPSS software was used to randomly allocate them into four groups: sedentary group (A-Sed), moderate-intensity continuous training group (A-MICT), and high-intensity interval training group (A-HIIT). Then, the mice were subjected to a 4-week program of sedentary, MICT, or HIIT accordingly. There were 8 mice in each group. The mice were raised in the room under a 12h/12h light-dark cycle with appropriate humidity (45-55%) and temperature (22±2°C), with *ad libitum* access to chow diet and water.

## Exercise training program

Our programs were modified from Rolim’s research [3]. After a 1-week adaptive feeding period, mice were acclimatized to the treadmill for 3 consecutive days. Then, they experienced an exhaustive running test to obtain the initial maximal running capacity. Mice in Young and A-Sed groups were fed normally without any exercise training for the entire duration of the experiment.The training plan was made according to the maximal speed. Briefly, the MICT program was a 40-minute at an intensity of 65%~70% maximum velocity and ended by relaxation phase till the end. The program of HIIT was 85%~90% maximum velocity for 2 min high-intensity exercise and then at 30% of maximum speed for 2 min low-intensity exercise. After several high-low cycles for 40 min, the mice underwent a relaxation phase until the end of the training as depicted in the diagram below. All mice experienced 60 min running exercise per day, 5 days per week according to the training plan for 4 weeks. Mice that could not complete the exhaustive running test or were injured in the exercise program were excluded. However, no mice were excluded during the whole experiment.


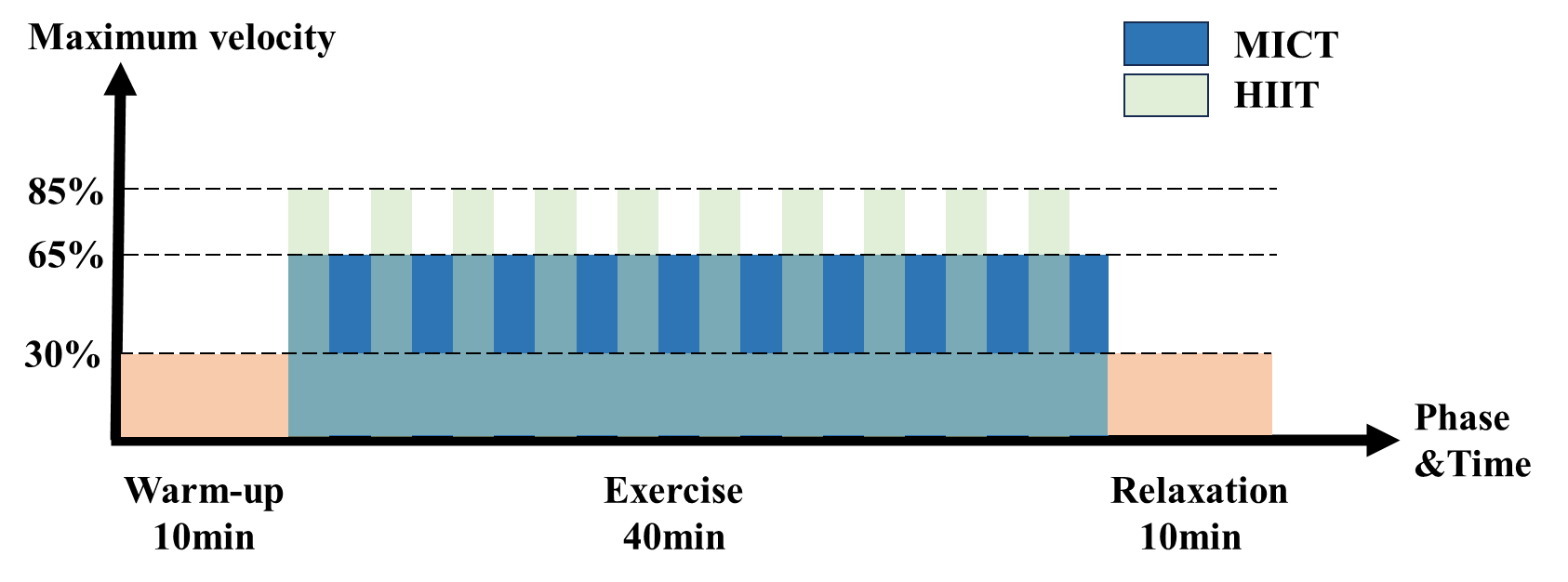


## Maximal running test

The maximal velocity of running was measured by a treadmill (Zhenghua, Anhui, CN). Initially, the running speed was set at 10 m/min with a slope angle of 0° and then increased by 2 m/min maintaining the slope angle at 0° throughout the process. When the mouse was continuously received stimulus for more than 10 seconds and did not return to the track, it was judged to be exhausted and removed from the treadmill. The running time and speed were recorded at the same time.

## Forelimb grip strength test

The grip strength of the forelimb was measured by an electronic dynamometer (ZP-100, AIGU, Hongkong, CN). The mouse was prompted to grasp the horizonal bar attached to the dynamometer in front of it. Then the tail of the mouse was pulled back parallel to the dynamometer and the mouse resisted this tension by gripping the bar with its forelimb. When the mouse released its grip due to the increasing force, it produced a maximum grip strength, and this value was recorded. The measurements were repeated 3 times for each mouse, and the average of these measurements was considered the maximal strength of the forelimb.

## Hanging grid test

The mice were placed on a grid of 100 cm×50 cm (small grid size, 2 cm×2 cm; thickness, 3 mm), and a soft cushion with a certain thickness was placed under the grid. The distance from the grid to the cushion was about 50 cm. When a mouse was placed at the center of the grid, the grid was turned over, hanging the mouse upside down. The time taken for the mouse to fall onto the cushion was recorded. Each mouse was tested for 3 times. The interval between tests was more than 30 min. Tthe average value of these three times was considered the hanging time of the mouse.

## Tissue processing

After the training, all mice were sacrificed to collect samples for further experiments. We first weighed the mice, then anaesthetized them by intraperitoneally injecting pentobarbital sodium. The gastrocnemius (GA) muscles were weighed, collected and partially frozen at -80℃. The other was fixed in 4% PFA, dehydrated and embedded with paraffin. 5 μm sections of gastrocnemius bellies slices were prepared for further experiments.

## Wheat germ agglutinin (WGA) staining

Muscle tissue was washed in PBS and then fixed in 4% PFA. The sections were then washed again to remove excess liquid. Next, the muscle was incubated with WGA-conjugates (L4895, Sigma, USA) as well as 4, 6-diamidino-2-phenylindole (DAPI, G1012, Servicebio, Wuhan, Hubei, CN) in the dark. **Four random fields of view in the lateral head of GA were selected to analyse**. Each field of view contained approximately 180-300 muscle fibers. The cross-sectional area (CSA) of fibers in these fields was averaged to obtain the muscle fiber CSA for each mouse. Images were captured using a fluorescence microscope (Leica, DM3000 LED, Wetzlar, Germany), and the CSA of myofibers was calculated by Image J.

## Immunofluorescence staining of paraffin section

First, the sections of GA were fixed in 4% PFA, then dehydrated and embedded in paraffin. After cutting the muscles into sections about 5μm thick, dewaxing and rewatering the sections were performed to further the experiment. For fiber typing, the paraffin sections were treated with PBS/3% BSA/10% donkey serum and permeabilized in PBS/0.1% Triton X-100 (GC204003, Servicebio) at room temperature. Subsequently, the sections were incubated with the slow skeletal myosin heavy chain (GB111857-100, Servicebio) and the fast myosin skeletal heavy chain (GB112130-100, Servicebio) antibodies at 4℃ overnight. The secondary antibodies were diluted in PBS/3% BSA/10% donkey serum and incubated with the sections for 1h. To measure the areas of the slow and the fast muscle fibers, images were obtained by a fluorescence microscope (Leica) and analyzed using Image J.

## Transmission electron microscope (TEM)

Gastrocnemius muscles were fixed with 2.5% glutaraldehyde containing 1% osmic acid. Then the samples were washed using 0.1mol/L phosphate buffer and dehydrated using a gradient acetone. Next, the samples were embedded and solidified, first at 37℃ for 12 hours and then at 60℃ for 24 hours. Thereafter, 50-100 nm sections obtained by slicing were examined using a transmission electron microscope (Tecnai G2 Spirit, FEI, USA).

# Cell experiments design

## Sample size (repetitions) calculation and statistical power

PASS software was used to calculate sample size of cellular experiments. The dependent experimental repetitions for our ANOVA study was calculated using standard power calculations with α = 0.05 and a power of 0.8. Based on previous study[4], we estimated the mean differences in the SA-β-gal staining area are 2.5%, 2.5%, 12.5%, and 7.5% with the standard deviation of 0.5% in Ctrl, s*iEef1e1*, d-gal, d-gal+s*iEef1e1* groups. As a result, 2 repetitions are needed, and we set at least 3 repetitions for cellular experiments.

## Cell treatments

C2C12 mouse myoblast obtained from a licensed Institute were cultured in Dulbecco's Modified Eagle's Medium (DMEM) with 10% fetal bovine serum and 1% penicillin/streptomycin (Gibco, New York, USA). We placed the cells in a cell incubator containing 5% CO_2_ with the temperature maintained at 37℃. The investigators were blinded to the group allocation during the detection experiment as well as during the analysis. The experiments were designed in detail as follows:

(1) The cells in the logarithmic growth phase were seeded in a 6-well plate at a density of 1×10^5^ cells per well. When they reached 70% confluency, the cells were treated with different dosage of D-gal (0, 10g/L, 20g/L, 40g/L, Sigma, USA) for 48h with the purpose of gaining the most appropriate concentration. This part was prepared for SA-β-Gal staining, cell count kit-8 (CCK8), western blot or RT-PCR.

(2) After being seeded in a 6-well plate, cells were subjected to several treatments for 24h: negative control siRNA, si*Eef1e1* transfection, overexpression plasmid or 500ng/ml recombinant EEF1E1 (Sinobiological, Beijing, CN) with 100nM bafilomycin A1 (Sigma) or vehicle control. Upon completion of the interventions, the cells were harvested for western blot or RT-PCR analysis.

(3) After cell adhesion, si*Eef1e1*, overexpression plasmid, or recombinant EEF1E1 were used to knock out or overexpression EEF1E1 at 50% confluency. To test the effect of aging, cells were treated with the most suitable dose of D-gal for 48h. To gain insight into the influence of treatments on differentiation, the differentiation medium was changed every 2 days. The cells were harvested for western blot, RT-PCR, SA-β-gal staining, or immunofluorescence staining.

## siRNA transfection

C2C12 cells were transfected with either siRNA or negative control siRNA (5μM/well) using Lipo3000 (L3000008, Invitrogen, USA) after adhesion. The negative control and specific siRNA were purchased from RiboBio Co.Ltd. The target sequence of si*Eef1e1* was GGCAGATTCCAGTTCTACA.

## SA-β-gal staining

According to the manual, we detected the activity of senescence-associated β-galactosidase (SA-β-gal) staining using the SA-β-gal staining kit (G1073, Servicebio). Briefly, cells subjected to different interventions were fixed using the fixation solution provided in the kit. After fixing, we rinsed the cells with a PBS buffer solution to remove any residual fixation solution. Subsequently, the cells were incubated with SA-β-gal and cultured in a 37°C incubator without CO_2_. Following staining, the cells exhibited a blue color when observed under an optical microscope. Three random fields of view were averaged to analyze. Each group was repeated five times.

## Myotube differentiation

Myoblasts were cultured in growth medium with different interventions. When they reached 80% confluency, the growth medium was replaced with differentiation medium containing 2% horse serum and 1% penicillin/streptomycin (P/S) in DMEM. The medium was changed every 2 days, and MyHC staining was performed to visualize the morphology of myotubes. Three random fields of view were averaged to analyze. Each group was repeated five times.

## Cell cycle assessment

1×10^5 cells per well were seeded in a 6-well plate for flow cytometry-based cell cycle analysis. After transfection and D-gal treatment, cells were trypsinized, collected, washed with PBS, and then centrifuged to remove the supernatant. The cells were fixed in pre-cooled 75% ethanol and incubated at 4℃ for 2 hours or more. Afterward, the ethanol was removed by centrifugation, and the cells were washed again with PBS to eliminate any residual ethanol. Finally, the cells were stained with a solution containing staining buffer, RNase A, and propidium iodide (PI) at 37℃ in the dark. After a 30-minute incubation, the cells were analyzed using a flow cytometer (NL3000, Cytek, CA, USA).

## Cell immunofluorescence staining

The adherent cells were fixed using 4%PFA and blocked and permeabilized with PBS/0.1% Triton X-100 (GC204003, Servicebio). Subsequently, the cells were incubated with antibodies for Lysosomal associated membrane protein 3 (LAMP3) and microtubule-associated protein light chain 3 (LC3) (Proteintech) respectively. After counterstaining nuclei with DAPI, capturing the images with a fluorescence microscope (Leica, Germany).

# Western blot procedures

Skeletal muscle tissues or myoblasts were used to extract protein. The samples were lysed with pre-cooled radioimmunoprecipitation assay (RIPA) buffer (Beyotime, Nanjing, China) as well as phenylmethanesulfonyl fluoride (PMSF; Beyotime). Next, the concentration of protein was measured using a Bicinchoninic acid (BCA) Protein Assay kit (Beyotime). Subsequently, the tissues or cells were processed by sodium dodecyl sulfate-polyacrylamide gel electrophoresis (SDS-PAGE) and transferred onto polyvinylidene difluoride (PVDF) membranes (Millipore, USA) followed by blocked in non-fat milk for 2h. **At this stage, the transferred protein could be reacted** **with primary antibodies against galactosidase beta 1 (GLB1), p16, p21, p53, atrogin1, muscle RING-finger protein-1 (MuRF1), myogenic differentiation antigen (MyoD), myosin heavy chain II (MyHC II), EEF1E1, SIRT1, adenosine 5‘-monophosphate (AMP)-activated protein kinase (AMPK), Unc-51-like-kinase (ULK), p-ULK (Ser-556), p62, microtubule-associated proteins 1A/1B light chain 3B (LC3), and glyceraldehyde 3-phosphate dehydrogenase (GAPDH), all sourced from Proteintech, Rosemont, USA. Additionally, antibodies against phosphorylated AMPK (Thr-172) and myogenin were obtained from Abcam, Cambridge, UK.** Overnight at 4℃, the samples were incubated with the corresponding secondary antibodies for 1h at room temperature. After being washed in TBST, the band were exhibited using a gel documentation system (Bio-Rad, Hercules, CA, USA).

# RT-PCR procedures

RNA was extracted using Trizol, followed by cDNA synthesis and RT-PCR using the PrimeScriptTM RT Reagent Kit with gDNA Eraser and SYBR Green PCR Master Mix (TaKaRa, Japan), respectively. After preparing the reaction mixture, the samples were placed in the instrument and subjected to the following program: denaturation at 95 °C for 30 seconds, followed by 40 cycles of amplification at 95 °C for 30 seconds and 60°C for 34 seconds, and final extension at 95 °C for 15 seconds, 60°C for 60 seconds, and 95 °C for 15 seconds. The specific primers used in this experiment are listed in the table.

| **Gene name** | **Sequence** | **Product length** |
| --- | --- | --- |
| M-EEF1E1 | F GGCCTCCACCGCTTTATAGT | 78bp |
|  | R GTGACAAAACCAGCGAGACAC |  |
| M-MyoD | F CATAGACTTGACAGGCCCCG | 92bp |
|  | R GCAGGTCTGGTGAGTCGAAA |  |
| M-Myogenin | F TACAGACGCCCACAATCTGC | 98bp |
|  | R AGTTGGGCATGGTTTCGTCT |  |
| M-GAPDH | F GCGACTTCAACAGCAACTCCC | 122bp |
|  | R CACCCTGTTGCTGTAGCCGTA |  |

#

# Protein-protein docking

The protein structure were selected from the PDB protein database: EEF1E1 (PDB：3VOQ) and SIRT1 (PDB：3VOQ). We investigated protein-protein dimer complex and the SIRT1 was set as receptor protein. EEF1E1 was docked into the receptor protein using ZDOCK. The PDBePISA was used to select docking model to verify optimal conformation (www.ebi.ac.uk/msd-srv/ssm/). Intermolecular forces and protein-protein interaction interfaces for the docking complex models were detected and analyzed using PyMol.

# Statistical analysis

The statistics are presented as Mean ± standard deviation (SD) and analyzed using R software. Investigators were always blinded to group allocation. When comparing two group means, Independent *t* test was used. For more than two groups, ANOVA with Bonferroni multiple comparisons test was used. The statistical method used for each experiment is indicated in each figure legend. *P* > 0.05 is considered non-significant (ns). *P* < 0.05 is considered significant.

Reference:

[1] Seldeen KL, Lasky G, Leiker MM, et al, High Intensity Interval Training Improves Physical Performance and Frailty in Aged Mice, J Gerontol a-Biol. 2018; 73 (4):429-437.

[2] Yang X, Li X, Yu N, et al, Proteomics and beta-hydroxybutyrylation Modification Characterization in the Hearts of Naturally Senescent Mice, Mol Cell Proteomics. 2023; 22 (11):100659.

[3] Rolim N, Skardal K, Hoydal M, et al, Aerobic interval training reduces inducible ventricular arrhythmias in diabetic mice after myocardial infarction, Basic Res Cardiol. 2015; 110 (4): 44.

[4] Yang YF, Yang W, Liao ZY, et al, MICU3 regulates mitochondrial Ca(2+)-dependent antioxidant response in skeletal muscle aging, Cell Death Dis. 2021; 12 (12):1115.

# ARRIVE checklist

|  |  | Reporting Item | Page Number |
| --- | --- | --- | --- |
| **Essential 10** |  |  |  |
| Study design | #1a | Give details of the groups being compared, including control groups. If no control group has been used, the rationale should be stated. | 2-3 |
| Study design | #1b | Give details of the experimental unit (e.g., a single animal, litter, or cage of animals). | 2-3 |
| Sample size | #2a | Specify the exact number of experimental units allocated to each group, and the total number in each experiment. Also indicate the total number of animals used. | 2 |
| Sample size | #2b | Explain how the sample size was decided. Provide details of any a priori sample size calculation, if done. | 2 |
| Inclusion and exclusion criteria | #3a | Describe any criteria used for including or excluding animals (or experimental units) during the experiment, and data points during the analysis. Specify if these criteria were established a priori. If no criteria were set, state this explicitly. | 3 |
| Inclusion and exclusion criteria | #3b | For each experimental group, report any animals, experimental units, or data points not included in the analysis and explain why. If there were no exclusions, state so. | 3 |
| Inclusion and exclusion criteria | #3c | For each analysis, report the exact value of n in each experimental group. | 2-3 |
| Randomisation | #4a | State whether randomisation was used to allocate experimental units to control and treatment groups. If done, provide the method used to generate the randomisation sequence. | 2 |
| Randomisation | #4b | Describe the strategy used to minimise potential confounders such as the order of treatments and measurements, or animal/cage location. If confounders were not controlled, state this explicitly. | 2 |
| Blinding | #5 | Describe who was aware of the group allocation at the different stages of the experiment (during the allocation, the conduct of the experiment, the outcome assessment, and the data analysis). | 12 |
| Outcome measures | #6a | Clearly define all outcome measures assessed (e.g., cell death, molecular markers, or behavioural changes). | 4 |
| Outcome measures | #6b | For hypothesis-testing studies, specify the primary outcome measure, i.e., the outcome measure that was used to determine the sample size. | 2 |
| Statistical methods | #7a | Provide details of the statistical methods used for each analysis, including software used. | 12 |
| Statistical methods | #7b | Describe any methods used to assess whether the data met the assumptions of the statistical approach, and what was done if the assumptions were not met. | 12 |
| Experimental animals | #8a | Provide species-appropriate details of the animals used, including species, strain and substrain, sex, age or developmental stage, and, if relevant, weight. | 2 |
| Experimental animals | #8b | Provide further relevant information on the provenance of animals, health/immune status, genetic modification status, genotype, and any previous procedures. | 2 |
| Experimental procedures | #9a | For each experimental group, including controls, describe the procedures in enough detail to allow others to replicate what was done, how it was done, and what was used. | 2-6 |
| Experimental procedures | #9b | Timing and frequency of procedures | 3 |
| Experimental procedures | #9c | Where procedures were carried out (including detail of any acclimatisation periods). | 2-3 |
| Experimental procedures | #9d | Rationale for procedures | 2-4 |
| Results | #10a | For each experiment conducted, including independent replications, report summary/descriptive statistics for each experimental group, with a measure of variability where applicable (e.g., mean and SD, or median and range). | 12 |
| Results | #10b | If applicable, for each experiment conducted, including independent replications, report the effect size with a confidence interval. | NA |
