# Supplementary material for: High‐Intensity Interval Training Mitigates Sarcopenia and Suppresses the Myoblast Senescence Regulator EEF1E1
Source: J Cachexia Sarcopenia Muscle. 2024 Sep 14;15(6):2574–85. doi: 10.1002/jcsm.13600 (PMC11634493; doi:10.1002/jcsm.13600)
Supplement: Supplementary file 4 — Table S1 Characteristics of Participants from the Randomized Crossover Trial (N = 10). Table S2. Characteristics of Cardiopulmonary Exercise Testing (N = 10). Table S3. Details of Differentially Expressed Plasma Proteins after MICT and HIIT. Table S4. Characteristics of Participants in the Sarcopenia Case–Control Study. Figure S1. Comparative Physiological Responses to High‐Intensity Interval Training (HIIT) and Moderate‐Intensity Continuous Training (MICT) in Sedentary Adults. MEC indicates maximal exercise capacity. Panels (A‐C) illustrate the responses in exercise intensity, heart rate, systolic blood pressure, and perceived exertion and dyspnea during HIIT; while Panels (D‐F) depict these responses during MICT. Figure S2. Participant Flow Diagram for the Randomized Crossover Trial. MICT refers to moderate‐intensity continuous training, while HIIT denotes high‐intensity interval training. Figure 3. Expression of Senescence Markers of Muscle in Aged Mice. Protein expressions of P16, P21, P53, and beta‐galactosidase (GLB1) in muscle tissues were analyzed (n = 3). Data are presented as the mean ± SD. An independent two‐tailed t‐test was used for comparisons between the young and aged groups. [file JCSM-15-2574-s003.docx]

Supplementary Document 4: Supplementary results

# **Supplementary Tables**

## sTable 1. Characteristics of Participants from the Randomized Crossover Trial (N=10).

|  | Total  (n=10) | MICT-HIIT group (n=5) | HIIT-MICT group (n=5) | *P*-value * |
| --- | --- | --- | --- | --- |
| Age, yrs | 32 (31–32) | 32 (30–34) | 32 (31–32) | 0.91 |
| Height, cm | 169 (162–176) | 170 (162–180) | 168 (141–177) | 0.99 |
| Body weight, kg | 66.8 (62.1–77.8) | 65.7 (59.3–74.1) | 71.1 (63.6–81.1) | 0.40 |
| Body mass index, kg/m^2^ | 23.2 (22.2–25.1) | 22.6 (21.4–24.3) | 24.0 (22.5–26.0) | 0.21 |
| Systolic blood pressure, mmHg | 116 (108–121) | 116 (107–128) | 115 (106–119) | 0.68 |
| Diastolic blood pressure, mmHg | 73 (64–82) | 78 (65–83) | 70 (64–79) | 0.53 |
| Waist circumference, cm | 80.0 (74.8–82.0) | 77.0 (74.0–81.5) | 82.0 (77.0–86.5) | 0.20 |
| Hip circumference, cm | 93.0 (89.8–95.0) | 90.0 (87.5–93.5) | 95.0 (92.0–98.5) | 0.11 |
| Total cholesterol, mmol/L | 4.61 (4.39–4.88) | 4.41 (3.96–5.07) | 4.62 (4.59–5.17) | 0.40 |
| Low-intensity lipoprotein, mmol/L | 2.92 (2.69–3.18) | 2.70 (2.54–3.18) | 3.02 (2.82–3.43) | 0.30 |
| High-intensity lipoprotein, mmol/L | 1.33 (1.05–1.44) | 1.32 (1.03–1.70) | 1.33 (1.09–1.48) | 0.99 |
| Triglycerides, mmol/L | 1.02 (0.85–1.45) | 1.09 (0.83–1.32) | 0.94 (0.84–2.10) | 0.83 |
| Fasting blood glucose, mmol/L | 5.08 (4.66–5.33) | 5.18 (5.00–5.35) | 4.67 (4.54–5.19) | 0.14 |
| Triiodothyronine, pmol/L | 4.87 (4.28–5.64) | 5.58 (4.07–5.68) | 4.69 (4.23–5.72) | 0.83 |
| Thyroxin, pmol/L | 12.61 (11.80–18.17) | 12.82 (11.61–18.97) | 12.40 (11.86–16.00) | 0.99 |
| TSH, μIU/ml | 1.33 (1.02–1.84) | 1.03 (0.83–2.27) | 1.55 (1.33–1.94) | 0.21 |
| White cell count, 10^9^/L | 5.65 (4.45–6.95) | 5.70 (5.05–7.55) | 5.40 (4.00–7.20) | 0.40 |
| Red cell count, 10^9^/L | 4.94 (4.68–5.18) | 4.85 (4.66–5.12) | 5.16 (4.67–5.24) | 0.60 |
| Hemoglobin, g/L | 153.0 (149.0–159.5) | 152.0 (148.5–159.0) | 154.0 (149.0–160.0) | 0.99 |
| Platelet count, 10^9^/L | 192.5 (158.5–217.3) | 210.0 (192.5–217.5) | 161.0 (137.5–204.5) | 0.14 |
| Plasma proteins, g/L | 73.6 (70.5–76.8) | 72.7 (67.1–77.5) | 74.5 (70.6–78.8) | 0.68 |
| Albumin, g/L | 47.2 (44.2–48.3) | 47.0 (42.3–47.8) | 48.0 (44.6–48.6) | 0.35 |
| Globulin, g/L | 27.1 (24.8–30.4) | 26.9 (24.1–29.8) | 27.1 (24.1–31.8) | 0.68 |
| Albumin/Globulin ratio | 1.65 (1.58–1.83) | 1.60 (1.60–1.85) | 1.70 (1.50–2.05) | 0.92 |

TSH indicates thyroid stimulating hormone. MICT-HIIT refers to the sequence of exercise where one begins with moderate-intensity continuous training and then transitions to high-intensity interval training. Conversely, HIIT-MICT denotes the exercise progression where one starts with high-intensity interval training and subsequently shifts to moderate-intensity continuous training. The data are presented as the median (interquartile range). * The difference between the MICT-HIIT and HIIT-MICT groups was determined by the Wilcoxon rank-sum test.

| Parameters | Values (N=10) |
| --- | --- |
| Peak workload, watts | 184 (144–201) |
| Peak heart rate, bpm | 173 (169–178) |
| Heart rate recovery in 1 min, bpm | 17 (10–25) |
| Peak systolic blood pressure, mmHg | 181 (159–195) |
| Peak diastolic blood pressure, mmHg | 85 (80–91) |
| Exercise ECG with ischemia presence, yes | 0 |
| Respiratory exchange ratio | 1.20 (1.14–1.23) |
| Peak oxygen consumption, ml/kg/min | 28.9 (27.3–38.1) |
| Oxygen consumption/pulse, ml/beat | 13.3 (10.7–14.5) |
| Peak ventilation, L/min | 70.2 (55.5–78.6) |
| Breath frequency, breath/min | 35 (31–36) |
| Breath reserve, % | 58.5 (50.0–62.5) |
| Minute ventilation/carbon dioxide production slope | 24.5 (23.4–26.0) |

## sTable 2. Characteristics of Cardiopulmonary Exercise Testing (N=10).

##

## sTable 3. Details of Differentially Expressed Plasma Proteins after MICT and HIIT.

| **Accession number** | **Protein descriptions** | **MW [KDa]** | **P-value** | **Fold changes** | **Exercise** |
| --- | --- | --- | --- | --- | --- |
| A0A0B4J1Y9 | Immunoglobulin heavy variable 3-72 | 13.2 | 0.0003 | 0.31 | HIIT |
| A0A0C4DH67 | Immunoglobulin kappa variable 1-8 | 12.5 | 0.0010 | 0.35 | HIIT |
| O43324 | Eukaryotic translation elongation factor 1 epsilon-1 | 19.8 | 0.0054 | 0.58 | HIIT |
| O60814 | Histone H2B type 1-K | 13.9 | 0.0001 | 0.01 | HIIT |
| P54108 | Cysteine-rich secretory protein 3 | 27.6 | 0.0249 | 0.52 | HIIT |
| P62328 | Thymosin beta-4 | 5.1 | 0.0039 | 0.42 | HIIT |
| P62877 | E3 ubiquitin-protein ligase RBX1 | 12.3 | 0.0057 | 0.65 | HIIT |
| Q76LX8 | A disintegrin and metalloproteinase with thrombospondin motifs 13 | 153.6 | 0.0134 | 0.67 | HIIT |
| A0A075B6H7 | Probable non-functional immunoglobulin kappa variable 3-7 | 12.8 | 0.0157 | 1.39 | HIIT |
| O75144 | ICOS ligand | 33.3 | 0.0305 | 1.56 | HIIT |
| P02671 | Fibrinogen alpha chain | 95.0 | 0.0032 | 2.02 | HIIT |
| P02775 | Platelet basic protein | 13.9 | 0.0005 | 1.78 | HIIT |
| P02776 | Platelet factor 4 | 10.8 | 0.0160 | 1.84 | HIIT |
| P05154 | Plasma serine protease inhibitor | 45.7 | 0.0292 | 1.73 | HIIT |
| P08294 | Extracellular superoxide dismutase [Cu-Zn] | 25.9 | 0.0142 | 1.65 | HIIT |
| P14923 | Junction plakoglobin | 81.7 | 0.0001 | inf | HIIT |
| P15924 | Desmoplakin | 331.8 | 0.0447 | 3.43 | HIIT |
| P49908 | Selenoprotein P | 43.2 | 0.0275 | 1.24 | HIIT |
| Q6EMK4 | Vasorin | 71.7 | 0.0194 | 1.27 | HIIT |
| Q6UX71 | Plexin domain-containing protein 2 | 59.6 | 0.0381 | 1.23 | HIIT |
| Q9BY67 | Cell adhesion molecule 1 | 48.5 | 0.0494 | 1.60 | HIIT |
| P04066 | Tissue alpha-L-fucosidase | 53.7 | 0.0403 | 0.64 | MICT |
| P08779 | Keratin, type I cytoskeletal 16 | 51.3 | 0.0284 | 0.45 | MICT |
| P12109 | Collagen alpha-1(VI) chain | 108.5 | 0.0001 | 0.01 | MICT |
| P15144 | Aminopeptidase N | 109.5 | 0.0461 | 0.77 | MICT |
| P24592 | Insulin-like growth factor-binding protein 6 | 25.3 | 0.0252 | 0.68 | MICT |
| P61769 | Beta-2-microglobulin | 13.7 | 0.0185 | 0.68 | MICT |
| Q12913 | Receptor-type tyrosine-protein phosphatase eta | 145.9 | 0.0006 | 0.55 | MICT |
| Q9H299 | SH3 domain-binding glutamic acid-rich-like protein 3 | 10.4 | 0.0309 | 0.63 | MICT |
| A0A075B6J2 | Probable non-functional immunoglobulin lambda variable 2-33 | 12.7 | 0.0001 | 2.40 | MICT |
| A0A087WSX0 | Immunoglobulin lambda variable 5-45 | 13.2 | 0.0212 | 1.62 | MICT |
| A0A0B4J1U7 | Immunoglobulin heavy variable 6-1 | 13.5 | 0.0001 | inf | MICT |
| A0A0C4DH29 | Immunoglobulin heavy variable 1-3 | 13.0 | 0.0146 | 1.69 | MICT |
| O00151 | PDZ and LIM domain protein 1 | 36.1 | 0.0496 | 1.38 | MICT |
| P01031 | Complement C5 | 188.3 | 0.0059 | 1.78 | MICT |
| P01833 | Polymeric immunoglobulin receptor | 83.3 | 0.0013 | 1.82 | MICT |
| P01871 | Immunoglobulin heavy constant mu | 49.4 | 0.0291 | 1.51 | MICT |
| P02745 | Complement C1q subcomponent subunit A | 26.0 | 0.0451 | 1.64 | MICT |
| P02746 | Complement C1q subcomponent subunit B | 26.7 | 0.0071 | 1.79 | MICT |
| P02747 | Complement C1q subcomponent subunit C | 25.8 | 0.0067 | 1.84 | MICT |
| P04114 | Apolipoprotein B-100 | 515.6 | 0.0238 | 1.54 | MICT |
| P04275 | von Willebrand factor | 309.3 | 0.0500 | 1.32 | MICT |
| P05062 | Fructose-bisphosphate aldolase B | 39.5 | 0.0309 | 1.90 | MICT |
| P07358 | Complement component C8 beta chain | 67.0 | 0.0432 | 1.36 | MICT |
| P07602 | Prosaposin | 58.1 | 0.0010 | inf | MICT |
| P0DOX5 | Immunoglobulin gamma-1 heavy chain | 49.3 | 0.0172 | 1.62 | MICT |
| P12821 | Angiotensin-converting enzyme | 149.7 | 0.0001 | inf | MICT |
| P12955 | Xaa-Pro dipeptidase | 54.5 | 0.0395 | 1.51 | MICT |
| P13521 | Secretogranin-2 | 70.9 | 0.0317 | 1.41 | MICT |
| P26038 | Moesin | 67.8 | 0.0001 | inf | MICT |
| P33908 | Mannosyl-oligosaccharide 1,2-alpha-mannosidase IA | 73.0 | 0.0006 | 2.06 | MICT |
| P80748 | Immunoglobulin lambda variable 3-21 | 12.4 | 0.0057 | 1.69 | MICT |
| Q07954 | Prolow-density lipoprotein receptor-related protein 1 | 504.6 | 0.0001 | inf | MICT |
| Q8NBP7 | Proprotein convertase subtilisin/kexin type 9 | 74.3 | 0.0417 | 1.50 | MICT |
| Q9BXR6 | Complement factor H-related protein 5 | 64.4 | 0.0093 | 1.82 | MICT |
| Q9HDC9 | Adipocyte plasma membrane-associated protein | 46.5 | 0.0386 | 1.42 | MICT |
| P05451 | Lithostathine-1-alpha | 18.7 | 0.0147 | 0.64 | MICT/HIIT |
| P0DOX4 | Immunoglobulin epsilon heavy chain | 60.3 | 0.0063 | 0.58 | MICT/HIIT |
| A0A075B6P5 | Immunoglobulin kappa variable 2-28 | 13.0 | 0.0104 | 1.63 | MICT/HIIT |
| P02675 | Fibrinogen beta chain | 55.9 | 0.0038 | 2.56 | MICT/HIIT |
| P02679 | Fibrinogen gamma chain | 51.5 | 0.0017 | 2.22 | MICT/HIIT |
| P81605 | Dermcidin | 11.3 | 0.0004 | 2.48 | MICT/HIIT |
| Q86SQ4 | Adhesion G-protein coupled receptor G6 | 136.7 | 0.0001 | 2.62 | MICT/HIIT |
| Q9HCN6 | Platelet glycoprotein VI | 36.9 | 0.0449 | 1.61 | MICT/HIIT |

## HIIT, high-intensity interval training; MICT, moderate-intensity continuous training.

## sTable 4. Characteristics of Participants in the Sarcopenia Case-Control Study.

|  | Control (N=42) | Sarcopenia (N=42) | *P* Value |
| --- | --- | --- | --- |
| Men, (n) % | 22 (52.4) | 22 (52.4) | 0.99 |
| Women, (n)% | 20 (47.6) | 20 (47.6) | 0.99 |
| Age, years | 74 (68­–80) | 75 (69–81) | 0.45 |
| Body weight, kg | 65.3 (57.3–68.9) | 53.5 (48.1–56.7) | <0.001 |
| Height, cm | 158.8 (153.9–166.1) | 160.8 (155.8–163.3) | <0.001 |
| BMI, kg/m^2^ | 24.8 (23.3–26.5) | 20.8 (19.3–22.5) | <0.001 |
| Waist circumference, cm | 86.0 (80.8–91.3) | 76.5 (70.8–84.0) | <0.001 |
| Hip circumference, cm | 95.0 (92.0–100.0) | 89.0 (86.0–91.3) | <0.001 |
| Medical history, (n) % |  |  |  |
| Hypertension | 17 (40.48) | 13 (30.95) | 0.49 |
| Type 2 Diabetes | 5 (11.91) | 6 (14.29) | 0.99 |
| Coronary artery disease | 1 (2.38) | 2 (4.76) | 0.99 |
| Appendicular skeletal muscle mass, kg | 19.1 (14.8–21.9) | 14.7 (13.3–16.7) | <0.001 |
| Left upper limb | 2.1 (1.7–2.5) | 1.7 (1.5–2.0) | <0.001 |
| Right upper limb | 2.2 (1.8-2.6) | 1.8 (1.6–2.0) | <0.001 |
| Left lower limb | 7.3 (5.6–8.5) | 5.7 (5.1–6.4) | <0.001 |
| Right lower limb | 7.4 (5.6–8.6) | 5.7 (5.1–6.5) | <0.001 |
| Skeletal muscle index | 7.12 (6.12–7.87) | 5.67 (5.51–6.30) | <0.001 |
| Left calf circumference, cm | 35.0 (33.4–37.0) | 32.0 (30.9–33.0) | <0.001 |
| Right calf circumference, cm | 35.0 (33.5–37.0) | 31.4 (30.0–32.5) | <0.001 |
| Physical Function |  |  |  |
| Hand grip strength*, kg | 29.4 (22.9–35.7) | 23.0 (20.1–26.2) | <0.001 |
| 6-meter walk, m/s | 1.07 (0.99–1.13) | 0.84 (0.64–0.91) | <0.001 |
| Chair stand test (30 seconds), repetition | 20 (19–25) | 18 (14–19) | <0.001 |

Continuous variables are presented as median (IQR), and categorical variables as count number (percentage). The difference between the control and sarcopenia groups is evaluated by Wilcoxon rank sum test for continuous variables, and by Fisher’s exact test for categorical variables.

* The values of hand grip strength were measured using the participants’ dominant hand.

# **Supplementary Figures**

**sFig 1. Comparative Physiological Responses to High-Intensity Interval Training (HIIT) and Moderate-Intensity Continuous Training (MICT) in Sedentary Adults.** MEC indicates maximal exercise capacity. Panels (A-C) illustrate the responses in exercise intensity, heart rate, systolic blood pressure, and perceived exertion and dyspnea during HIIT; while Panels (D-F) depict these responses during MICT.

**sFig 2. Participant Flow Diagram for the Randomized Crossover Trial.** MICT refers to moderate-intensity continuous training, while HIIT denotes high-intensity interval training.


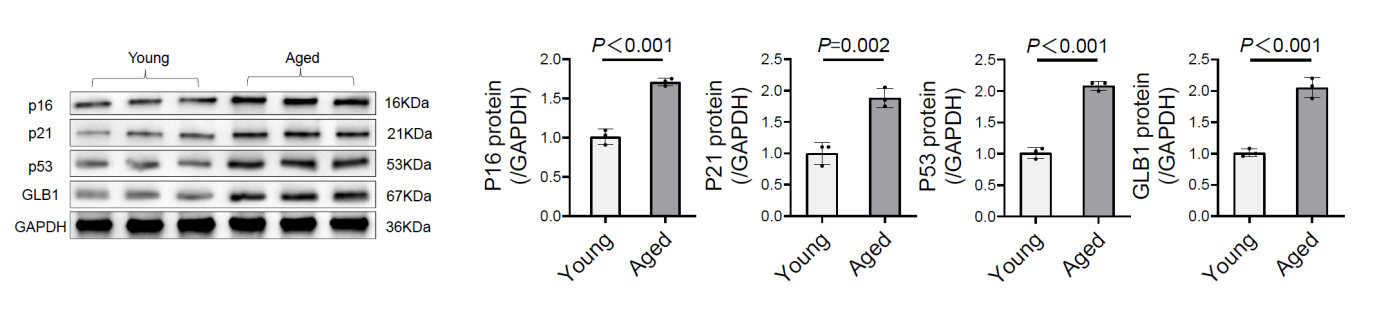


## sFig 3. Expression of Senescence Markers of Muscle in Aged Mice. Protein expressions of P16, P21, P53, and beta-galactosidase (GLB1) in muscle tissues were analyzed (n = 3). Data are presented as the mean ± SD. An independent two-tailed t-test was used for comparisons between the young and aged groups.
